# Discovery of potent and versatile CRISPR–Cas9 inhibitors engineered for chemically controllable genome editing

**DOI:** 10.1093/nar/gkac099

**Published:** 2022-02-21

**Authors:** Guoxu Song, Fei Zhang, Chunhong Tian, Xing Gao, Xiaoxiao Zhu, Dongdong Fan, Yong Tian

**Affiliations:** CAS Key Laboratory of RNA Biology, Institute of Biophysics, Chinese Academy of Sciences, Beijing 100101, China; University of Chinese Academy of Sciences, Beijing 100049, China; CAS Key Laboratory of RNA Biology, Institute of Biophysics, Chinese Academy of Sciences, Beijing 100101, China; University of Chinese Academy of Sciences, Beijing 100049, China; CAS Key Laboratory of RNA Biology, Institute of Biophysics, Chinese Academy of Sciences, Beijing 100101, China; University of Chinese Academy of Sciences, Beijing 100049, China; CAS Key Laboratory of RNA Biology, Institute of Biophysics, Chinese Academy of Sciences, Beijing 100101, China; CAS Key Laboratory of RNA Biology, Institute of Biophysics, Chinese Academy of Sciences, Beijing 100101, China; CAS Key Laboratory of RNA Biology, Institute of Biophysics, Chinese Academy of Sciences, Beijing 100101, China; CAS Key Laboratory of RNA Biology, Institute of Biophysics, Chinese Academy of Sciences, Beijing 100101, China; University of Chinese Academy of Sciences, Beijing 100049, China

## Abstract

Anti-CRISPR (Acr) proteins are encoded by many mobile genetic elements (MGEs) such as phages and plasmids to combat CRISPR–Cas adaptive immune systems employed by prokaryotes, which provide powerful tools for CRISPR–Cas-based applications. Here, we discovered nine distinct type II-A anti-CRISPR (AcrIIA24–32) families from *Streptococcus* MGEs and found that most Acrs can potently inhibit type II-A Cas9 orthologs from *Streptococcus* (SpyCas9, St1Cas9 or St3Cas9) in bacterial and human cells. Among these Acrs, AcrIIA26, AcrIIA27, AcrIIA30 and AcrIIA31 are able to block Cas9 binding to DNA, while AcrIIA24 abrogates DNA cleavage by Cas9. Notably, AcrIIA25.1 and AcrIIA32.1 can inhibit both DNA binding and DNA cleavage activities of SpyCas9, exhibiting unique anti-CRISPR characteristics. Importantly, we developed several chemically inducible anti-CRISPR variants based on AcrIIA25.1 and AcrIIA32.1 by comprising hybrids of Acr protein and the 4-hydroxytamoxifen-responsive intein, which enabled post-translational control of CRISPR–Cas9-mediated genome editing in human cells. Taken together, our work expands the diversity of type II-A anti-CRISPR families and the toolbox of Acr proteins for the chemically inducible control of Cas9-based applications.

## INTRODUCTION

Bacteria and archaea are under constant threat of infections by mobile genetic elements (MGEs), such as viruses (phages) and plasmids (1,2). To defend against varied attacks by invasive MGEs, prokaryotes have evolved multiple immune mechanisms, including restriction–modification systems, abortive infection and CRISPR (clustered regularly interspaced short palindromic repeats)–Cas (CRISPR-associated) systems (3,4). The immune function of CRISPR–Cas generally involves three stages, adaptation, expression and interference, allowing host cells to remember, recognize and destroy re-invading foreign genetic elements (5). CRISPR–Cas systems are categorized into two classes, six types and multiple subtypes, which are multi-subunit effector class 1 (types I, III and IV) and single effector class 2 (types II, V and VI) (6). The class 2 type II DNA-targeting nuclease Cas9 is the first Cas effector utilized for genome editing, which assembled with CRISPR RNA (crRNA) and *trans*-activating crRNA (tracrRNA) or a single-guide RNA (sgRNA; engineered by fusing crRNA and tracrRNA) to specifically target foreign nucleic acids flanked by a protospacer adjacent motif (PAM) (7,8). The Cas9 from *Streptococcus pyogenes* (SpyCas9) is the most commonly used Cas effector and has been developed as a series of powerful tools for genome engineering (9,10).

In recent years, the natural inhibitors of CRISPR–Cas systems, called anti-CRISPR (Acr) proteins, have been developed into the off-switches for CRISPR–Cas systems (11,12). Acr proteins are the products of the evolutionary arms race between phages and host cells and are encoded by diverse MGEs to combat CRISPR–Cas systems in prokaryotes (13,14). To date, 88 distinct Acr families have been reported, inhibiting type I, II, III, V and VI CRISPR–Cas systems (15,16). Notably, 23 AcrIIA families have been discovered via various methods, such as ‘self-targeting’, ‘guilt-by-association’, ‘phage-first’ and other approaches (17–27). AcrIIA proteins exhibit diverse inhibitory mechanisms against Cas9, which can be subsequently classified into three strategies, including crRNA loading interference, DNA binding prevention and DNA cleavage blockage (24,28,29). For example, AcrIIA2 and AcrIIA4 mimic DNA and then block Cas9 binding to DNA, while AcrIIA6 induces Cas9 dimerization to reduce Cas9 binding affinity for DNA (30,31). Recent studies also showed that AcrIIA14 inhibits DNA cleavage of Cas9 by affecting the activity of HNH domain, and AcrIIA15 binds directly to Cas9 with the interference of sgRNA loading (23,32). Although these diverse inhibitory mechanisms of Acrs have enriched our knowledge about the roles of Acrs in the phage–host arms race, a large number of unknown Acr proteins may still exist, while only a few Acr proteins have been elucidated in depth on inhibitory mechanism. This hinders not only our understanding of the evolutionary battle between phages and hosts in CRISPR–Cas biology, but also the development of novel Acr-based applications in genome editing (11). Considering the off-target effects of Cas9, it is of particular interest to develop Acr proteins as useful genome editing tools for modulating Cas9 activity to address issues of efficacy and safety (12). However, applicable Acr proteins and strategies as efficient tools to control Cas9 activities in gene editing are still limited (12).

In this study, we utilized the widespread *AcrIIA6* as an initial marker gene to discover nine new *anti-CRISPR* genes (*AcrIIA24–32*) along with three *aca* (Acr-associated) genes (*aca11–13*). We found that AcrIIA24–32 families are mainly distributed in *Streptococcus* and most Acrs exhibit robust inhibitions on type II-A Cas9 orthologs from *Streptococcus* (SpyCas9, St1Cas9 and St3Cas9) in bacterial and human cells. We also found that AcrIIA24–32 can exploit versatile abilities to inhibit Cas9 through diverse mechanisms. Importantly, AcrIIA25.1 and AcrIIA32.1 can inhibit both DNA binding and DNA cleavage of SpyCas9, exhibiting unique anti-CRISPR characteristics. These two proteins were further developed as chemically inducible anti-CRISPR (iAcr) variants for post-translational control of CRISPR–Cas9-mediated genome editing in human cells. Our work expands our understanding of CRISPR–Cas biology and phage–host interactions and provides strategies for controllable regulation of Cas9-based applications.

## MATERIALS AND METHODS

### Microbes

*Escherichia coli* (TOP10 or Mach1-T1, Biomed) strains were used for plasmid amplification and interference assays. *Escherichia coli* (T7 Express, Biomed) strains were used for protein expression and phage plaque assays. *Escherichia coli* were routinely (unless otherwise indicated) cultured at 37°C in lysogeny broth (LB) medium with appropriate antibiotics (when required): ampicillin (50 μg/ml), kanamycin (50 μg/ml) or chloramphenicol (25 μg/ml).

### Cell lines

HEK293T and HEK293T-BFP cells were cultured in DMEM (Gibco) medium supplemented with 10% (vol/vol) fetal bovine serum (FBS, Gibco) at 37°C and 5% CO_2_ in an incubator. U2OS cells were cultured in McCoy’s 5A (modified) medium (Gibco) supplemented with 10% FBS at 37°C and 5% CO_2_ in an incubator.

### Bioinformatics analysis

BLASTp program was used to search for AcrIIA6 (accession: AVO22749.1) homologs in the nonredundant protein database to manually examine the possible novel *acr* and *aca* genes from neighboring candidate genes. A gene was designated as an *aca* according to previous methods (33) by the following criteria: (i) directly upstream or downstream of an Acr homolog in the same orientation; (ii) containing a DNA-binding domain predicted by HHpred of MPI Bioinformatics Toolkit (34); and (iii) this gene can associate with more than two distinct types of Acrs.

BLASTp searches using *aca or acr* genes were conducted to screen *acr* gene candidates using the ‘guilt-by-association’ method, which were subject to further validation through biochemical analysis. A gene was considered as the Acr candidate by the following criteria: (i) small protein size (<300 amino acids); (ii) direct upstream or downstream of Acr or Aca proteins in the same orientation; and (iii) fusion feature of Acr with Aca as a more confident marker.

For phylogenetic analysis of Acr proteins, homologous protein sequences of Acrs were obtained by BLASTp program using the nonredundant protein database. Sequences with high homology (*E*-value <0.001, query coverage >70%) were determined to generate distance trees based on BLAST using the fast minimum-evolution tree method, 0.85 maximum sequence difference and the Grishin (protein) distance model (24). Further labels were edited using the MEGA X (35) and Illustrator (Adobe).

### Plasmid interference assays in *E. coli*

Plasmids (pB001–pB008 and pB017–pB050) used in the interference assays were designed based on our previous report (36) and are listed in Supplementary Table S5. DNA sequences encoding Acr proteins were synthesized by Biomed or GenScript and ligated into the pBAD24 vector. The spacer sequences of Cas9 orthologs for targeting pT are shown in Supplementary Figure S2A and Supplementary Table S5. Plasmids were transformed into *E. coli* using the CaCl_2_ heat-shock procedure as described previously with slight modifications (37). Briefly, *E. coli* TOP10 or Mach1-T1 strains carrying Acr plasmids were cultured overnight in LB medium with 0.2% arabinose and then used as competent cells for subsequent transformation with 25 ng of pT and 25 ng of Cas9 (with matching spacer or mismatching spacer) plasmids. After recovery for 2 h in LB medium with 0.2% arabinose, cells were plated on LB agar with antibiotics (50 μg/ml ampicillin, 50 μg/ml kanamycin and 25 μg/ml chloramphenicol) and inducers (1 mM IPTG and 0.2% arabinose) and incubated at 37°C for 24–32 h. Clones were photographed using a gel scanner (Tanon 3500) and counted via ImageJ software. Inhibitory activity of each Acr was shown in percentage by calculating the ratio of cfu (colony forming units) between *E. coli* transformed with Cas9 plasmid with matching spacer and that of the mismatching spacer, which was the average of at least three biological replicates.

### Phage plaque assays

Plasmids (pB009–pB016 and pB018–pB032) used in phage plaque assays were designed based on our plasmid interference assays in *E. coli* and are listed in Supplementary Table S5. Phage plaque assays were performed according to previous reports with some modifications (38,39). Briefly, *E. coli* (T7 Express, Biomed) cells were co-transformed with a plasmid expressing Cas9–sgRNA combinations targeting phage T4 and a compatible plasmid encoding Acr proteins. Both Cas9 plasmid for nontargeting phage T4 and an empty pBAD24 plasmid (no Acr) served as controls. The *E. coli* containing both Acr and Cas9 plasmids were cultured in LB medium supplemented with antibiotics (50 μg/ml ampicillin and 25 μg/ml chloramphenicol) and grown overnight at 37°C. The next morning, overnight cultures were inoculated in fresh LB medium with antibiotics and grown at 37°C for 2 h. Subsequently, 1 mM IPTG was added to induce the expression of Cas9 proteins. Two hours later, 0.2% arabinose was added to induce the expression of Acr proteins. After another 2 h, 200 μl culture was mixed with 4 ml molten top LB agar (0.7%) supplemented with 10 mM MgSO_4_ and poured over the prewarmed bottom LB agar (1.5%) plates containing 10 mM MgSO_4_, 0.2% arabinose, 1 mM IPTG and both antibiotics. Next, 10-fold serial dilutions of phage T4 lysate were spotted on the lawn surface. The plates were incubated overnight at 37°C and photographed using a gel scanner (Tanon 3500).

### Protein expression and purification

DNA sequences encoding St1Cas9, St3Cas9 or Acr proteins were incorporated into the pET28a vector for protein expression in *E. coli* (T7 Express, Biomed) (pC003–pC017, Supplementary Table S5). *Escherichia coli* cells were routinely induced for protein expression in LB medium with 1 mM IPTG for 16 h at 18°C supplemented with 50 μg/ml kanamycin. Cells were harvested and resuspended in lysis buffer (50 mM Tris–HCl, pH 8.0, 10 mM imidazole, 0.5 mM TCEP–NaOH and 500 mM NaCl) supplemented with 1 mM PMSF and lysozyme. After sonication and centrifugation, the supernatant of cells was bound to Ni-NTA agarose (QIAGEN), and bound protein was eluted with 500 mM imidazole. Amicon Ultra centrifugal filter (Millipore) was used to concentrate proteins and exchange buffer into storage buffer [20 mM HEPES–NaOH, pH 7.5, 5% (v/v) glycerol, 300 mM NaCl and 1 mM DTT]. For Acr proteins, the second round of Ni-NTA purification was conducted to isolate untagged Acr proteins after incubation with tobacco etch virus protease (Sangon Biotech) overnight at 4°C. To minimize protein degradation, we prepared aliquots of all purified proteins stored at −80°C, and avoided repeated freezing and thawing.

### *In vitro* DNA cleavage assays

All sgRNAs in the assays were prepared using *in vitro* T7 transcription kit (Invitrogen) according to the manufacturer’s manual, and the transcription templates were generated with linearized sgRNA plasmids (pC018–pC023, Supplementary Table S5). SpyCas9 protein was purchased from Invitrogen.

For Figure 3, and Supplementary Figures S4 and S5, the plasmid pC002 was constructed and further linearized through NotI restriction endonuclease (NEB) (Supplementary Table S5). For Figure 3 and Supplementary Figure S4, cleavage reactions were conducted in a total volume of 10 μl with NEBuffer 3.1, along with Cas9 proteins (500 nM), sgRNA (500 nM), target DNA substrate (30 ng/μl) and excess Acr proteins (10 μM). In addition, we conducted assays with SpyCas9 ribonucleoprotein (RNP) (256 nM) and Acr titrations (0, 128, 256, 512, 1024, 2048, 4096 and 8192 nM) in Supplementary Figure S5. For Figure 3 and Supplementary Figure S5, Cas9 protein was complexed with sgRNA for 10 min at 37°C. The Acr proteins were then added and incubated at room temperature for another 20 min. For Supplementary Figure S4, Cas9 protein was incubated with Acr protein at room temperature for 20 min. The sgRNA was then added and incubated for 10 min at 37°C. Subsequently, for all assays, target DNA was added and incubated for 10 min at 37°C. Reactions were stopped after the addition of 1 μl of Proteinase K. Products were analyzed on 1% agarose/1× TAE (Tris–acetate–EDTA) gels, which were visualized by the gel scanner (Tanon 3500).

For Figure 6H and I, and Supplementary Figure S8E, fluorescently labeled substrate DNA was used for cleavage assays, which was prepared by annealing the synthetic oligos of target and nontarget strands labeled with Cy5 or Cy3 (Supplementary Table S3). The process of cleavage assays is shown in Figure 6G. Briefly, Cas9 proteins (500 nM) and sgRNA (500 nM) were mixed to form Cas9 RNP complex for 10 min at 37°C in 1× binding buffer [20 mM Tris–HCl, pH 7.6, 150 mM KCl, 5 mM EDTA, 5 mM MgCl_2_, 1 mM DTT, 5% (v/v) glycerol, 50 μg/ml heparin, 0.01% Tween 20 and 100 μg/ml BSA] to abrogate the cleavage activity of Cas9. Then, Acr proteins (10 μM) and substrate DNA (50 nM) were added in a different order, and incubated for 20 min at room temperature, respectively. Subsequently, MgCl_2_ (10 mM) was added to restore the DNA cleavage activity of Cas9, and then incubated for another 20 min at room temperature. The reactions were stopped through the addition of Gel Loading Buffer II (Invitrogen) and incubated at 85°C for 6 min. Products were analyzed on 12% denaturing PAGE gel and visualized by Typhoon 7000 (GE).

### Construct of intein–Acr plasmids

DNA sequences encoding Acr proteins (AcrIIA4, AcrIIA5, AcrIIA25.1 and AcrIIA32.1) were cloned into the pCDNA3.1 vector for expression in human cells. Intein 37R3-2 sequence (40) was synthesized and inserted into the described positions of Acr proteins for construction of intein–Acr plasmids (pM043–pM052, Supplementary Table S5).

### T7 endonuclease 1 assay

Plasmids (pM001–pM024, pM038–pM042, pM049 and pM051) expressing Cas9 orthologs, Acr or iAcr proteins, and sgRNAs used in T7 endonuclease 1 (T7E1) assays are listed in Supplementary Table S5. Target sequences in *AAVS1*, *EMX1* and *DYRK1A* loci and primer sequences for PCR amplification are provided in Supplementary Table S2. For Figure 4, HEK293T cells cultured in the 24-well plate were transfected with 1 μg of Cas9 plasmid, 0.5 μg of sgRNA plasmid and 0.5 μg of Acr plasmid per well, using the Lipofectamine LTX reagent (Invitrogen) according to the manufacturer’s protocol. For Figure 7D–F, HEK293T cells were transfected with 1 μg of Cas9 plasmid, 0.5 μg of sgRNA plasmid, and 0.5 or 0.25 μg of Acr or iAcr plasmid per well in the 24-well plate, with or without 4-hydroxytamoxifen (4-HT, 1 μM, Selleck S7827). At 72 h post-transfection, genomic DNA from cells was extracted with the DNeasy Blood and Tissue Kit (QIAGEN) and amplified for PCR with Q5 High-Fidelity Polymerase (NEB). PCR products mixed with NEBuffer 2 were denatured and annealed before T7E1 (NEB) was added and incubated at 37°C. Samples were fractionated in a 3% agarose/1× TAE gel. T7 bands were quantified using the ImageJ software. The efficiency of genome editing in mammalian cells was calculated with the following formula: indel (%) = 100 $ \times {\rm{\ }}( {1 - \sqrt {( {1 - {\rm fraction}_{{{\rm cleaved}}}} )} } )$.

### Fluorescence imaging for telomeric foci

Plasmids (pM009–pM012, pM025–pM027, pM032 and pM033) encoding Nme_dCas9-(sfGFP)_3_, Spy_dCas9-(mCherry)_3_, Spy_Cas9-(mCherry)_3_, their respective sgRNAs targeting telomeres and Acrs (AcrIIC1, AcrIIA4 and AcrIIA5) were used from our previous study (36). Vectors (pM013–pM024, pM028–pM031, pM034 and pM035) expressing St1_dCas9-(mCherry)_3_, St1_Cas9-(mCherry)_3_, St3_dCas9-(mCherry)_3_, St3_Cas9-(mCherry)_3_, their respective sgRNAs targeting telomeres and other Acrs are listed in Supplementary Table S5. For imaging, U2OS cells were cultured on 15-mm glass-bottom dishes (Electron Microscopy Sciences) in 24-well plates and co-transfected with 60 ng of each (d)Cas9 plasmid, 300 ng of each sgRNA-telomere plasmid and 300 ng of individual Acr plasmid using Lipofectamine LTX reagent (Invitrogen) according to the manufacturer’s manual. At 24 h post-transfection, cells were fixed with 4% paraformaldehyde (Beyotime) and imaged using a Nikon A1R+ confocal microscope with a 60× oil objective lens.

The ‘blind’ experiments were performed according to the previous studies (41). One experimenter coded the cells from each condition by labeling numbers. Another experimenter observed and scored the cells under the microscope, who did not know these conditions. For quantifications, only the cells expressing TagBFP and mCherry fluorescence as well as Nme_dCas9-(sfGFP)_3_ telomeric foci were assessed in the presence or absence of co-localizing S**_(d)Cas9-(mCherry)_3_ telomeric foci.

### Electrophoretic mobility shift assays

RNA electrophoretic mobility shift assays (EMSAs) were performed by incubating Cas9 protein (256 nM) and sgRNA (256 nM) in the presence or absence of Acrs (5 μM) with the indicated order in figure legends. Reactions were incubated in 1× binding buffer [20 mM Tris–HCl, pH 7.6, 150 mM KCl, 5 mM EDTA, 5 mM MgCl_2_, 1 mM DTT, 5% (v/v) glycerol, 50 μg/ml heparin, 0.01% Tween 20 and 100 μg/ml BSA]. The sgRNAs and Acr proteins were added in different order and incubated for 10 min at 37°C, respectively. Samples were analyzed on 6% Tris–borate–EDTA (TBE) polyacrylamide gels and visualized using SYBR Gold (Invitrogen) stain by Typhoon 7000 (GE). We performed DNA EMSAs as previously described (42). Briefly, Cas9–sgRNA complexes were incubated in 1× binding buffer for 10 min at 37°C with the indicated concentration in figure legends. Subsequently, Acr proteins with various concentrations were added and incubated for 20 min at room temperature, and then 20 nM fluorescently labeled substrate DNA (Cy5-labeled target strand) was added to the mix followed by incubation for 10 min at 37°C. In a parallel experiment, fluorescently labeled substrate DNA was added and incubated for 10 min at 37°C before Acr proteins with various concentrations were added and incubated for 20 min at room temperature. Samples were analyzed by biphasic polyacrylamide (the upper half of the gel is 6% and the lower half of the gel is 12%)/0.5× TBE gel electrophoresis. Gels were visualized by Typhoon 7000 (GE). All assays were conducted in triplicates.

### Next-generation sequencing

Gene editing efficiencies were assessed by next-generation sequencing (NGS) using a two-step PCR-based method. Briefly, genomic DNA from cells was extracted with the DNeasy Blood and Tissue Kit (QIAGEN) and amplified for first-step PCR with Q5 High-Fidelity Polymerase (NEB). Target sequences in *EMX1* and *DYRK1A* loci and primer sequences carrying 5′ Illumina sequencing adaptors for PCR amplification are provided in Supplementary Table S2. PCR products were purified using QIAquick PCR Purification Kit (Qiagen) and serve as template for second-step PCR with primer sequences carrying Illumina barcodes by Q5 High-Fidelity Polymerase (NEB). The PCR products were sequenced on an Illumina MiSeq machine by PE150 via commercial sequencing service (Tsingke Biotechnology) and the efficiency of genome editing was determined from the sequencing data using CRISPResso2 (43).

### PE-mediated BFP-to-GFP gene editing in HEK293T-BFP cells

First, we established HEK293T-BFP cells with a chromosomally integrated BFP at *AAVS1* locus (Supplementary Figure S9A) in HEK293T cells based on a previous report (44). Briefly, HEK293T cells were transfected with Cas9, AAVS1-targeting sgRNA plasmids and a donor vector containing homologous sequences and cassette sequences with *BFP* and *puromycin* resistant genes. HEK293T-BFP cells were selected by puromycin (2 μg/ml) treatment and flow cytometry (FACSAriaIII, BD).

To examine the activity of intein–Acr hybrids against Cas9 in human cells, we performed prime editing (PE)-mediated BFP-to-GFP gene editing in HEK293T-BFP cells in the presence or absence of intein–Acr variants under the condition of 4-HT treatment or not. The plasmid encoding prime editor PE2 was purchased from Addgene (#132775). The BFP-targeting pegRNA plasmid was constructed by synthesizing DNA sequences containing the target, sgRNA scaffold, PBS and RT template incorporated into the U6-sgRNA vector (Supplementary Figure S9C and Supplementary Table S5). HEK293T-BFP cells were cultured in 24-well plates prior to transfection with plasmids encoding prime editor PE2 (1 μg), BFP-targeting pegRNA (0.5 μg) and Acr (0.25 μg) per well using Lipofectamine LTX reagent (Invitrogen), with or without 4-HT (1 μM, Selleck S7827). At 72 h post-transfection, cells were collected and the percentage of GFP-positive cells by flow cytometry (FACSAriaIII, BD) was calculated.

## RESULTS

### Multiple AcrIIA proteins are discovered from *Streptococcus* MGEs

**Figure 1.**
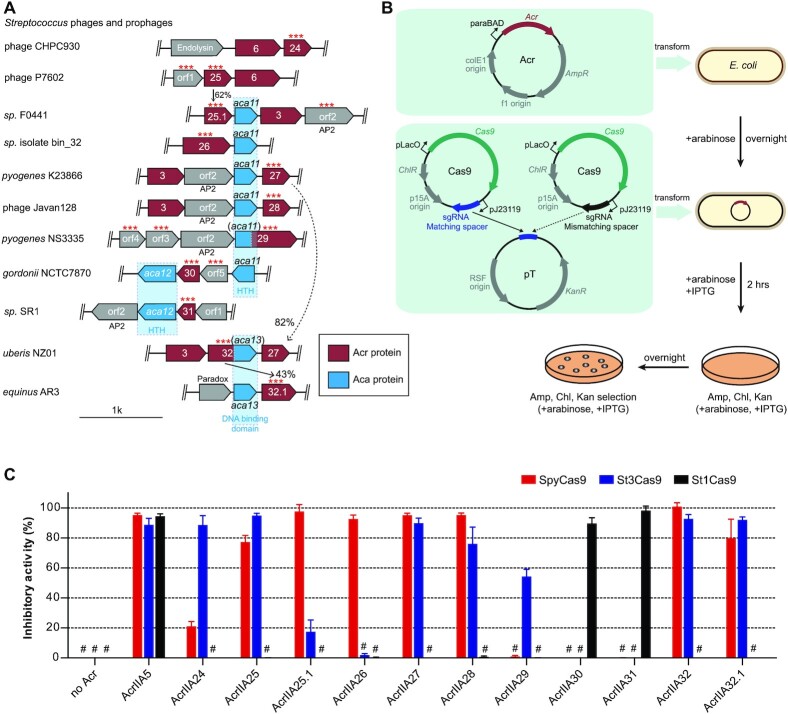
Identification and validation of nine AcrIIA families from *Streptococcus* MGEs. (**A**) Schematic view of *acr*, *aca* and relevant neighboring genes in the genome of *Streptococcus* phages and prophages (see Supplementary Figure 1A for full schematic). *Acr* genes are shown in red with numbers corresponding to AcrIIA numbers. Arrows indicate the relationship between *acr* loci with the percentage of protein sequence identity. *Aca* genes are shown in blue with numbers and their predicted domains are displayed below. Helix–turn–helix (HTH) and AP2 DNA binding motifs were detected by HHpred (see the ‘Materials and Methods’ section). Other neighboring genes are shown in gray and some known genes are annotated according to the NCBI website. Individual genes (***) were assayed for CRISPR–Cas9 inhibition in *E. coli*; orf, open reading frame. (**B**) Schematic view of designed plasmids and procedure of plasmid interference assays for Acr activity analysis in *E. coli*. AmpR, ampicillin resistance; KanR, kanamycin resistance; ChlR, chloramphenicol resistance. (**C**) Bar graphs show the calculated inhibitory activity of each Acr against type II-A Cas9 orthologs (SpyCas9, St1Cas9 and St3Cas9) in *E. coli*. Inhibitory activity of each Acr was shown in percentage by calculating the ratio of cfu between *E. coli* transformed with Cas9 plasmid with matching spacer and that of the mismatching spacer. Experiments were repeated at least three times. The ‘#’ sign represents ‘below detection limit of this assay’. Each bar represents the mean ± SEM.

Apart from SpyCas9, different Cas9 orthologs from *Streptococcus* genomes have also been developed as various gene editing tools recently (45,46). Many *Streptococcus* genomes exhibit a self-targeting phenomenon investigated by the self-target spacer searcher system (47). However, known Acrs cannot be matched in most of these genomes, suggesting that there may be multiple unknown Acrs in *Streptococcus* genomes. Acrs always cluster together within ‘defense islands’ in bacterial genomes, so it is possible to find Acrs by the ‘guilt-by-association’ method using known Acrs or Aca protein as the search marker according to a previous study (2). To identify potential Acrs within the *Streptococcus* MGEs, we conducted bioinformatics searches through the BLAST program using the widespread *AcrIIA6* gene as an initial query sequence (Figure 1A and Supplementary Figure S1A) (19). The relevant neighboring genes were taken into account as Acr or Aca candidates (see the ‘Bioinformatics analysis’ section for details) and were examined via a preliminary high-throughput screening approach by plasmid interference assays in *E. coli* for subsequent assays, which we called the ‘search-validation’ process. The validated *Acr* or *Aca* genes were used as markers to further expand the scope of bioinformatics searches, and the new candidate Acr proteins were investigated by another round of plasmid interference assays. Acr candidates were tested against the most widely used *Streptococcus* Cas9 systems, i.e. SpyCas9 and *S. thermophilus* Cas9 (CR1/St1Cas9 and CR3/St3Cas9) (48), using our established plasmid interference assays in *Escherichia coli* (Figure 1B) (36). In plasmid interference assays, DNA fragments encoding Cas9 and Acr proteins were cloned into compatible bacterial expression plasmids. The plasmid pT containing a protospacer was targeted by Cas9 plasmid with matching spacer, while another Cas9 plasmid with mismatching spacer served as a control (Supplementary Figure S2A). Inhibitory activity of each Acr was measured by calculating the ratio of cfu between *E. coli* transformed with Cas9 plasmid with matching spacer and that of the mismatching spacer (Figure 1C, and Supplementary Figures S1B and S2B). After several rounds of the ‘search-validation’ process, we screened ∼30 Acr candidates and identified nine distinct *anti-CRISPR* genes (named *AcrIIA24–32*) along with three *aca* genes (named *aca11–13*) from *Streptococcus* MGEs (Supplementary Table S1). We found that AcrIIA24–32 exhibited similar inhibition on SpyCas9 and St3Cas9, but the inhibition on St1Cas9 was quite different (Figure 1C and Supplementary Figure S2B). This result may be due to the fact that SpyCas9 and St3Cas9 are closer in sequence homology, compared with St1Cas9. Among 11 Acr proteins (addition of two Acr orthologs: AcrIIA25.1 and AcrIIA32.1) we examined, 5 Acr proteins (AcrIIA25, AcrIIA27, AcrIIA28, AcrIIA32 and AcrIIA32.1) exhibited robust inhibitory activities on both SpyCas9 and St3Cas9. AcrIIA25.1 and AcrIIA26 potently inhibited SpyCas9, while AcrIIA24 and AcrIIA29 inactivated St3Cas9 with various degrees. Additionally, both AcrIIA30 and AcrIIA31 strongly inhibited St1Cas9 activity (Figure 1C). However, other Acr candidates (orf1–17) from neighboring genes had no inhibitory activity on SpyCas9, St1Cas9 and St3Cas9 in our experiments (Supplementary Figure S1B). In addition, we noticed that several proteins (orf1, orf12 and orf15) are toxic when expressed in *E. coli*, so it is difficult to determine whether these proteins are Acr proteins or not. In sum, our data indicated that multiple Acrs from AcrIIA24–32 can potently inactivate type II-A Cas9 orthologs from *Streptococcus* in *E. coli* (Table 1).

**Table 1.** Summary of anti-CRISPR proteins reported in this study and their inhibitory activities

Acr	Strain	Accession number	Bacterial Assays			Human Assays		
			SpyCas9	St1Cas9	St3Cas9	SpyCas9	St1Cas9	St3Cas9
AcrIIA24	*Streptococcus* phage CHPC930	AZF92405.1	+	-	+++	-	ND	+++
AcrIIA25	*Streptococcus* phage P7602	ARU14017.1	++	-	+++	+	ND	++
AcrIIA25.1	*Streptococcus sp*. F0441	WP_009730541.1	+++	-	+	+++	ND	ND
AcrIIA26	*Streptococcus sp*. isolate bin_32	RKW07596.1	+++	-	-	+++	ND	ND
AcrIIA27	*Streptococcus pyogenes* K23866	WP_003055855.1	+++	-	+++	+++	ND	+++
AcrIIA28	*Streptococcus* phage Javan128	QBX23430.1	+++	-	+++	+++	ND	+++
AcrIIA29	*Streptococcus pyogenes* NS3335	WP_136281566.1	-	-	++	ND	ND	+
AcrIIA30	*Streptococcus gordonii* NCTC7870	WP_143877237.1	-	+++	-	ND	++	ND
AcrIIA31	*Streptococcus sp*. SR1	WP_033585134.1	-	+++	-	ND	+++	-
AcrIIA32	*Streptococcus uberis* NZ01	WP_199763731.1	+++	-	+++	+++	ND	+++
AcrIIA32.1	*Streptococcus equinus* AR3	WP_094140900.1	+++	-	+++	+++	ND	+++

20% < inhibitory activity < 50%, marks “+”; 50% < inhibitory activity < 80%, marks “++”; and inhibitory activity > 80% marks “+++”. ND, not determined.

To further determine the homology landscape of AcrIIA24–32, we performed comprehensive phylogenetic analyses based on BLAST results (Supplementary Figure S3). Our data showed that AcrIIA28, AcrIIA30 and AcrIIA32 homologs were rare and they were only distributed in several *Streptococcus* genomes or phages (Supplementary Figure S3E, G and I). In contrast, homologs of other Acr proteins were more widely distributed across *Streptococcus* MGEs. AcrIIA26, AcrIIA27, AcrIIA29 and AcrIIA31 families were observed in multiple strains of *Streptococcus*, such as *S. mitis*, *S. salivarius* and *S. pyogenes* (Supplementary Figure S3C, D, F and H). In addition, AcrIIA24 and AcrIIA25 homologs were found not only in strains of *Streptococcus* genomes, but also in various *Streptococcus* phages (Supplementary Figure S3A and B). These data showed that AcrIIA24–32 families are mainly distributed in *Streptococcus*, indicating a potential role for AcrIIA24–32 in arms race between phages and hosts in *Streptococcus*.

### AcrIIA24–32 are specific inhibitors of the *Streptococcus* CRISPR–Cas9 systems

Previous studies showed that some Acrs exhibit broad-spectrum inhibitions on diverse Cas9 orthologs, like AcrIIC1 and AcrIIA5 (36,42). We then asked whether AcrIIA24–32 exert broader inhibitions on other Cas9 orthologs. We examined the inhibitory activities of these Acr proteins against the Cas9 from *Neisseria meningitidis* (NmeCas9), a well-studied Cas9 ortholog belonging to type II-C CRISPR–Cas systems. Through the plasmid interference assays, our data showed that AcrIIA24–32 had no inhibition on NmeCas9 in *E.coli*, while AcrIIA5 can efficiently inhibit NmeCas9 (Supplementary Figure S2C).

**Figure 2.**
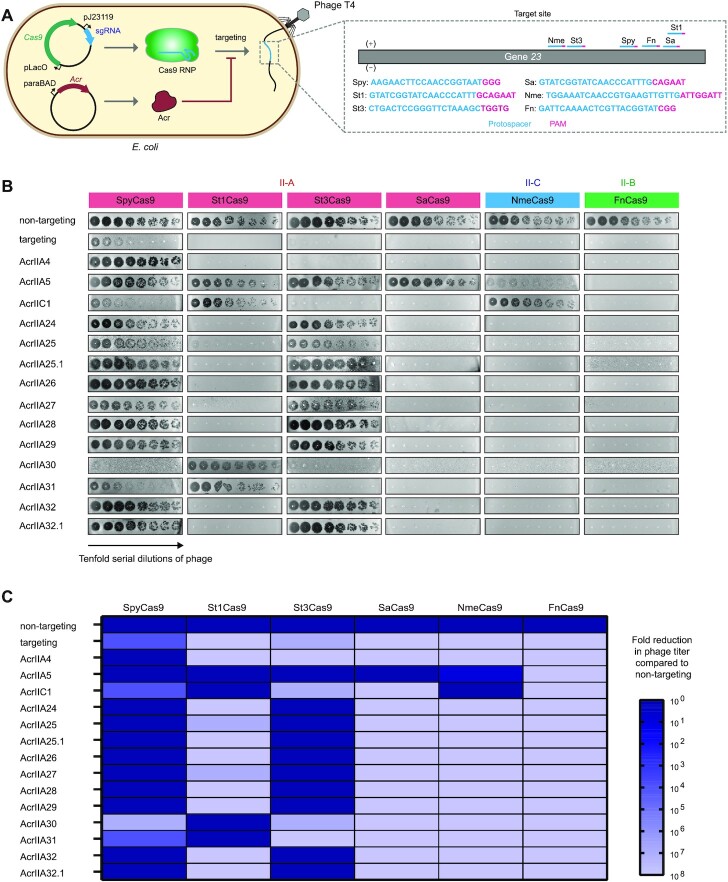
AcrIIA24–32 are specific inhibitors of the *Streptococcus* CRISPR–Cas9 system. (**A**) Schematic view of phage plaque assays to investigate whether AcrIIA24–32 have broad-spectrum activity on diverse type II-A, II-B and II-C Cas9 orthologs. *Escherichia coli* carrying plasmids for expressing Cas9, sgRNA and Acr were challenged with the CRISPR-targeted phage T4. The gene *23* of phage T4 was targeted by different Cas9 orthologs (abbreviated by strain names). (**B**) Phage plaque assays were performed with 10-fold serial dilutions of phage T4 (black circles) to assess the inhibition of different Acr proteins against diverse type II-A (SpyCas9, St1Cas9, St3Cas9 and SaCas9), II-B (FnCas9) and II-C (NmeCas9) Cas9 orthologs. (**C**) A matrix showing the inhibitory activity of Acrs against diverse Cas9 orthologs in phage plaque assays. The degree of blue represents the inhibitory activity of indicated Acrs against Cas9 orthologs (e.g. the cell with darkest blue indicates the given Acr with strong inhibition on Cas9). Values of this figure represent the mean of at least three biological replicates.

To further investigate whether AcrIIA24–32 are broad-spectrum inhibitors of Cas9, we performed phage plaque assays to examine the possible inhibitory activities of AcrIIA24–32 against diverse well-characterized Cas9 orthologs, including type II-A [SpyCas9, St1Cas9, St3Cas9 and *Staphylococcus aureus* Cas9 (SaCas9)], II-B [*Francisella novicida* Cas9 (FnCas9)] and II-C (NmeCas9) systems (Figure 2A). We used phage T4 to perform phage plaque assays. Although the cytosine hydroxymethylation and glucosylation of phage T4 DNA can block Cas9 targeting, some studies showed that Cas9 can effectively target and cleave the genome of phage T4 at some sites such as gene *23* (38,39,49). In our work, *E. coli* cells carrying Cas9 expression plasmids with a spacer targeting the gene *23* of phage T4 (nontargeting spacer as controls) were challenged with 10-fold serial dilutions of phage T4 in the presence or absence of Acrs. We found that all Cas9 orthologs successfully reduced the phage T4 plaque, while SpyCas9 displayed a slightly weaker efficiency to target phage T4, when compared to other Cas9 orthologs (Figure 2B). We speculated that SpyCas9 may be more sensitive to cytosine hydroxymethylation and glucosylation of the T4 DNA than other Cas9 orthologs. In addition, we found that AcrIIA30 and AcrIIA31 can specifically inhibit St1Cas9, while other Acrs can inhibit both SpyCas9 and St3Cas9 (Figure 2B and C). We also quantified the inhibitory activity of Acrs against diverse Cas9 orthologs in phage plaque assays. The results showed that AcrIIA30 and AcrIIA31 robustly inhibit St1Cas9, and other Acrs can strongly inhibit both SpyCas9 and St3Cas9. These proteins restored phage T4 replication to nearly the same levels as in the nontargeting control (Figure 2B and C). Our data also showed that AcrIIA24–32 displayed no detectable inhibitory activity on SaCas9, NmeCas9 and FnCas9. Through phage plaque assays, we confirmed that AcrIIA24–32 are bona fide anti-CRISPR proteins and these proteins are specific inhibitors of the *Streptococcus* CRISPR–Cas9 systems.

### AcrIIA24–32 inhibit type II-A Cas9 orthologs *in vitro*

**Figure 3.**
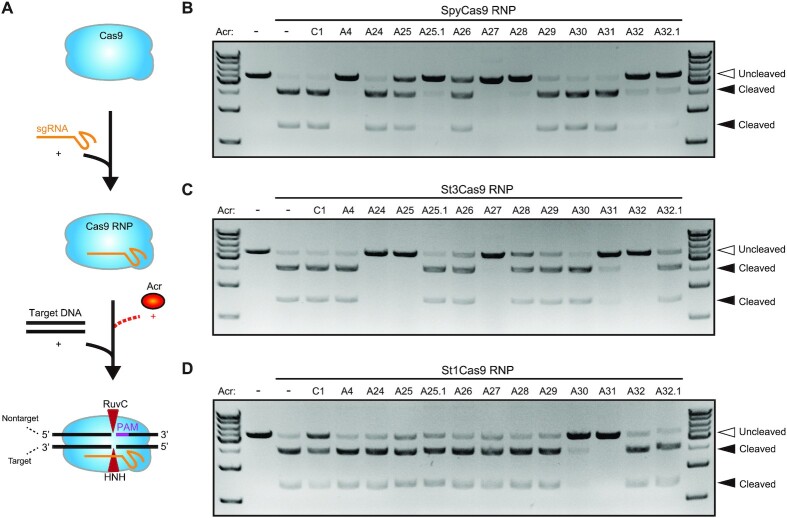
AcrIIA24–32 inhibit the *Streptococcus* type II-A Cas9 orthologs *in vitro*. (**A**) Schematic overview of DNA cleavage assays, in which Cas9 RNP was used to target a DNA substrate in the presence of Acr proteins. DNA cleavage assays using SpyCas9 (**B**), St3Cas9 (**C**) and St1Cas9 (**D**) RNPs to target a linearized plasmid DNA in the presence or absence of Acr proteins. Acr subtypes and numbers are indicated. A, AcrIIA; C, AcrIIC. Hollow arrowheads indicate the uncleaved linearized plasmid DNA. Solid arrowheads indicate cleaved products. The gel images shown for DNA cleavage assays are representative of three independent replicates.

In plasmid interference assays, some of AcrIIA24–32 proteins exhibited weak inhibitory activities on *Streptococcus* CRISPR–Cas9 systems in *E. coli* (Figure 1C). However, in phage plaque assays, AcrIIA24–32 exhibited robust inhibitory activities of these Acr proteins against type II-A Cas9 orthologs (SpyCas9, St1Cas9 and St3Cas9) (Figure 2B and C). To eliminate this discrepancy and confirm the inhibitory activities of AcrIIA24–32 against *Streptococcus* CRISPR–Cas9, we purified Cas9 and Acr proteins and performed DNA cleavage assays *in vitro* (Supplementary Figure S4A). The inhibitory activities of AcrIIA24–32 were measured by SpyCas9, St1Cas9 and St3Cas9 RNPs for targeting a linearized plasmid in the presence or absence of Acrs (Figure 3A and Supplementary Figure S4B). The results showed that SpyCas9 can be inhibited by AcrIIA25, AcrIIA25.1, AcrIIA26, AcrIIA27, AcrIIA28, AcrIIA32 and AcrIIA32.1 (Figure 3B). Other Acr proteins except AcrIIA25.1 and AcrIIA30 can inhibit St3Cas9, while AcrIIA24, AcrIIA25, AcrIIA27, AcrIIA31 and AcrIIA32 display stronger inhibitory activities against St3Cas9 (Figure 3C). In addition, only AcrIIA30 and AcrIIA31 can potently inactivate St1Cas9 *in vitro* (Figure 3D). The result about inhibitory activities of AcrIIA24–32 proteins in DNA cleavage assays is largely consistent with that in the plasmid interference assays. We speculated that discrepancies of Acr inhibitory activities among different assays may be due to the sensitivity of different experiments. Phage T4, as the virulent phage of *E. coli*, can utilize these proteins (including weak Acrs) to efficiently escape from CRISPR immunity. Thus, phage plaque assays are sensitive approaches to detect Acrs even displaying an enhanced inhibitory effect of Acrs.

We also analyzed the dose-dependent effect of Acr proteins against SpyCas9 using DNA cleavage assays with SpyCas9 RNP (256 nM) and Acr titrations (0, 128, 256, 512, 1024, 2048, 4096 and 8192 nM). The results showed that AcrIIA25.1, AcrIIA32.1 and AcrIIA28 can inhibit SpyCas9 at lower molar concentration (∼512 nM), but AcrIIA26 and AcrIIA27 require higher concentration (above 2048 nM) to inhibit SpyCas9, compared to AcrIIA4 control (∼1024 nM) (Supplementary Figure S5). In addition, we performed DNA cleavage assays, using pre-incubated apo-Cas9 with Acr proteins before sgRNA and target DNA were introduced into the reaction (Supplementary Figure S4C–F). We found no significant difference under these two reaction conditions, suggesting that AcrIIA24–32 mainly act on Cas9 RNP to impact the downstream function of Cas9 RNP.

### AcrIIA24–32 inhibit Cas9 ortholog-mediated gene editing in human cells

**Figure 4.**
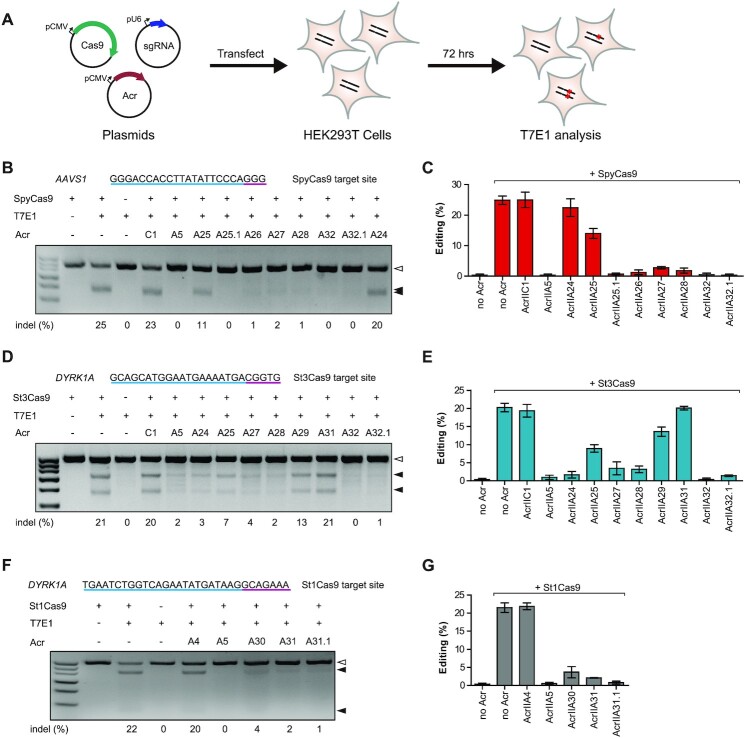
AcrIIA24–32 inhibit different Cas9-mediated gene editing in human cells. (**A**) Schematic view of T7E1 assay to examine Acr inhibition against Cas9 orthologs in HEK293T cells. Cells were co-transfected with plasmids encoding Cas9, sgRNA and Acr and subsequently analyzed through T7E1 assay. Representative gel images of T7E1 assay to manifest the inhibitory activities of Acrs against SpyCas9 (**B**), St3Cas9 (**D**) and St1Cas9 (**F**). The target sites of human *AAVS1* (targeted by SpyCas9) and *DYRK1A* (targeted by St1Cas9 and St3Cas9) are shown at the top of each gel and PAMs are highlighted in purple. Acr subtypes and numbers are indicated. A, AcrIIA; C, AcrIIC. Hollow arrowheads indicate the T7E1-undigested bands (unedited). Solid arrowheads indicate T7E1-digested bands (edited). The editing efficiencies [indel (%)] are labeled at the bottom of each lane. Quantification of gene editing efficiencies of SpyCas9 (**C**), St3Cas9 (**E**) and St1Cas9 (**G**) is shown in the presence of different Acrs. Error bars represent the mean ± SEM with three biological replicates.

Given the wide applications of various Cas9 orthologs in eukaryotic cells, we next examined whether the corresponding Acrs can inhibit Cas9 orthologs in human cells. HEK293T cells were co-transfected with plasmids encoding Cas9, genome-targeting sgRNAs and Acrs. The individual editing efficiency was then analyzed using T7E1 at 72 h post-transfection (Figure 4A). Human endogenous *AAVS1* and *DYRK1A* loci were designed for gene editing using type II-A Cas9 orthologs from *Streptococcus*.

Strikingly, we observed that multiple Acrs exhibited robust inhibitions on SpyCas9, St1Cas9 and St3Cas9 in human cells (Table 1). The SpyCas9 activity was nearly completely suppressed by AcrIIA25.1, AcrIIA26, AcrIIA27, AcrIIA28, AcrIIA32 and AcrIIA32.1 at levels comparable with a known potent inhibitor AcrIIA5 control, while AcrIIA25 only weakly inhibited SpyCas9 with 44% inhibitory activity on average (Figure 4B and C). In addition, St3Cas9-mediated gene editing can be inhibited with different degrees by multiple Acrs, including AcrIIA24 (average 91%), AcrIIA25 (average 56%), AcrIIA27 (average 83%), AcrIIA28 (average 84%), AcrIIA29 (average 33%), AcrIIA32 (average 98%) and AcrIIA32.1 (average 93%) (Figure 4D and E). We also found that the activity of St1Cas9 can be efficiently inhibited by AcrIIA30, AcrIIA31 and an AcrIIA31 ortholog (AcrIIA31.1) (Figure 4F and G). Thus, our results indicated that multiple Acrs from AcrIIA24–32 and their orthologs can potently inhibit *Streptococcus* Cas9 orthologs (SpyCas9, St1Cas9 and St3Cas9) in human cells.

### Diverse strategies are employed by Acrs to inactive Cas9 in human cells

**Figure 5.**
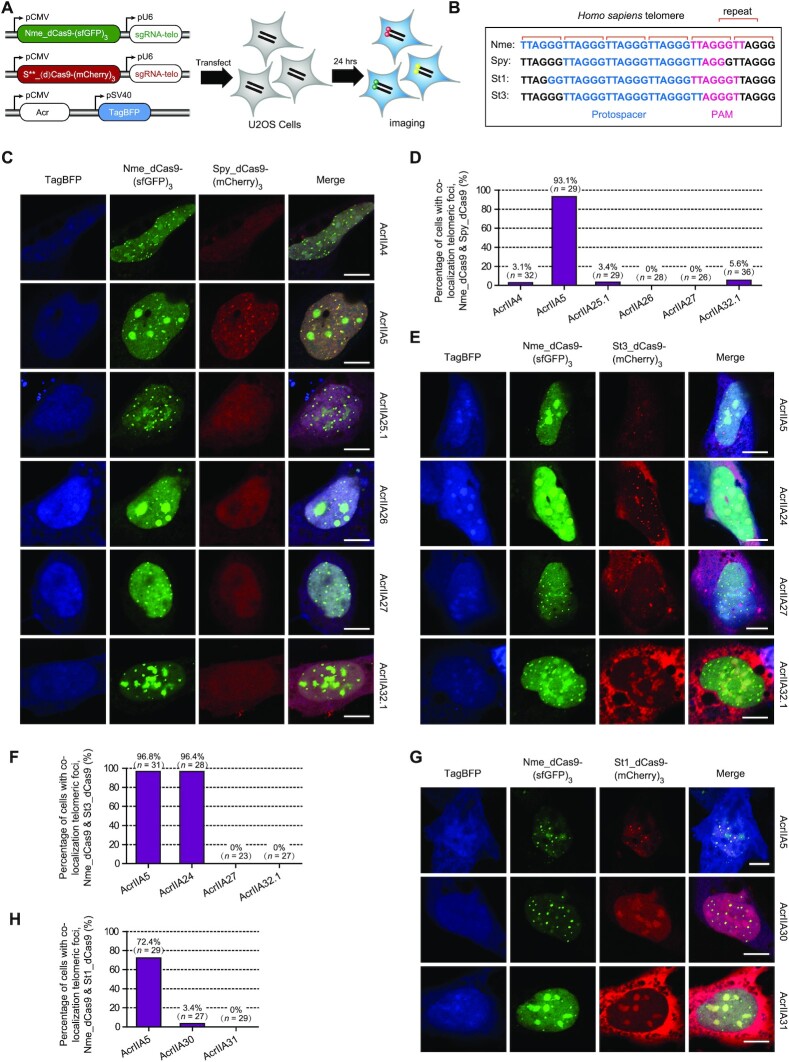
Diverse strategies employed by Acrs to disable Cas9 in human cells. (**A**) A schematic view of the plasmids designed for fluorescence localization of human telomeric foci to investigate the inhibitory strategy of Acrs against type II-A Cas9 orthologs. U2OS cells were co-transfected with plasmids encoding Cas9-fluorescent proteins, their respective sgRNAs targeting telomeres and Acr proteins (marked with the blue fluorescent protein TagBFP). S**_(d)Cas9-(mCherry)_3_ represents six plasmids used in this assay, including Spy_dCas9-(mCherry)_3_, Spy_Cas9-(mCherry)_3_, St1_dCas9-(mCherry)_3_, St1_Cas9-(mCherry)_3_, St3_dCas9-(mCherry)_3_ and St3_Cas9-(mCherry)_3_. (**B**) Diagrams showing targeted human telomeres in U2OS cells by Cas9 orthologs (Nme, Spy, St1 and St3) and their respective protospacer sequences. (**C**) Representative images of U2OS cells co-transfected with Nme_dCas9-(sfGFP)_3_, Spy_dCas9-(mCherry)_3_ and Acr plasmids. The fluorescent channels are shown at the top of the figure, and different Acr proteins are shown at the right of each row. The scale bars represent 10 μm. (**D**) Quantitation of Spy_dCas9-(mCherry)_3_ telomeric foci by calculating the percentage of cells with co-localization telomeric foci of Nme_dCas9-(sfGFP)_3_ and Spy_dCas9-(mCherry)_3_ in the presence of different Acr proteins. Foci were scored blind (see the ‘Materials and Methods’ for details). *n* = number of cells that were scored under each condition. Representative images of U2OS cells after transfection with St3_dCas9-(mCherry)_3_ (**E**) or St1_dCas9-(mCherry)_3_ (**G**), along with Nme_dCas9-(sfGFP)_3_ and different Acr plasmids. The scale bars represent 10 μm. Quantitation of St3_dCas9-(mCherry)_3_ (**F**) and St1_dCas9-(mCherry)_3_ (**H**) telomeric foci under each condition using the same method as in panel (D).

To investigate the inhibitory mechanisms of Acr proteins we discovered against Cas9, we selected AcrIIA24, AcrIIA25.1, AcrIIA26, AcrIIA27, AcrIIA30, AcrIIA31 and AcrIIA32.1 to perform the fluorescence imaging assays based on the co-localization imaging of Cas9 orthologs to human telomeric foci in U2OS cells (41,50) (Figure 5A and B). The anti-CRISPR effects of Acrs can be evaluated by telomeric DNA binding of mCherry-labeled *Streptococcus* Cas9 orthologs, using superfolder GFP-labeled NmeCas9 as a telomeric indicator.

First, we investigated the effects of potent inhibitors on SpyCas9 binding to DNA, including AcrIIA25.1, AcrIIA26, AcrIIA27 and AcrIIA32.1, along with AcrIIA4 and AcrIIA5 as controls. We observed the co-localization of Spy_dCas9-(mCherry)_3_ and Nme_dCas9-(sfGFP)_3_ to telomeric foci in cells with co-expressed AcrIIA5 or no Acr control plasmid, while the red telomeric foci formed by Spy_dCas9-(mCherry)_3_ were abolished by AcrIIA4 (Figure 5C and Supplementary Figure S6A). However, co-expression of AcrIIA25.1, AcrIIA26, AcrIIA27 or AcrIIA32.1 all resulted in the loss of red telomeric foci formation by Spy_dCas9-(mCherry)_3_ without affecting green telomeric foci formed by Nme_dCas9-(sfGFP)_3_ (Figure 5C). We then repeated the experiments in a blinded experimental setup and quantified the number of cells exhibiting Spy_dCas9-(mCherry)_3_ telomeric foci in the presence of different Acr proteins. We observed Spy_dCas9-(mCherry)_3_ telomeric foci in 93.1% of cells in the presence of AcrIIA5, while the Spy_dCas9-(mCherry)_3_ telomeric foci in cells expressing AcrIIA25.1, AcrIIA26, AcrIIA27, AcrIIA32.1 or AcrIIA4 control were rarely observed (Figure 5D). We further used the catalytically active Cas9 labeled with mCherry (Spy_Cas9-(mCherry)_3_) for cell imaging in U2OS cells. Consistent with previous results of Spy_dCas9-(mCherry)_3_ telomeric foci, AcrIIA25.1, AcrIIA26, AcrIIA27 and AcrIIA32.1 potently abrogated the formation of red telomeric foci by Spy_Cas9-(mCherry)_3_ (Supplementary Figure S6B and C). These results confirmed that AcrIIA25.1, AcrIIA26, AcrIIA27 and AcrIIA32.1 can strongly block the DNA binding by SpyCas9 in human cells.

Subsequently, to explore the mechanisms of potent inhibitors of St1Cas9 and St3Cas9, we established a similar fluorescence imaging system using St1Cas9 and St3Cas9 for targeting human telomeric foci in U2OS cells (Figure 5A and B). We observed the co-localization of St3_dCas9-(mCherry)_3_ or St1_dCas9-(mCherry)_3_ with Nme_dCas9-(sfGFP)_3_ to telomeric foci in cells co-expressed with AcrIIA5 or no Acr plasmid (Figure 5E and G, and Supplementary Figure S6A). AcrIIA24 had no effect on the co-localization of telomeric foci by St3_dCas9-(mCherry)_3_, while AcrIIA27, AcrIIA32.1, AcrIIA30 and AcrIIA31 can abolish red telomeric foci formed by St3Cas9 or St1Cas9 (Figure 5E and G). We also quantified the number of cells exhibiting St3_dCas9-(mCherry)_3_ or St1_dCas9-(mCherry)_3_ telomeric foci in the presence of different Acr proteins. We observed St3_dCas9-(mCherry)_3_ foci in 96.8% of cells expressing AcrIIA5 and in 96.4% of cells expressing AcrIIA24, while no cells expressing AcrIIA27 or AcrIIA32.1 exhibit St3_dCas9-(mCherry)_3_ foci (Figure 5F). In addition, St1_dCas9-(mCherry)_3_ foci were observed in 72.4% of cells expressing AcrIIA5, in 3.4% of cells expressing AcrIIA30 and in 0% of cells expressing AcrIIA31 (Figure 5H). We further constructed St3_Cas9-(mCherry)_3_ and St1_Cas9-(mCherry)_3_ plasmids containing catalytically active Cas9 labeled with mCherry. We found that AcrIIA27, AcrIIA32.1, AcrIIA30 and AcrIIA31 abolished red telomeric foci formed by St3_Cas9-(mCherry)_3_ or St1_Cas9-(mCherry)_3_, and no cells expressing these Acrs were observed with red telomeric foci when compared to AcrIIA5 control (Supplementary Figure S6D–G). In addition, AcrIIA24 had no effect on co-localization of telomeric foci by St3_Cas9-(mCherry)_3_ and Nme_dCas9-(sfGFP)_3_, and 88.5% of cells expressing AcrIIA24 exhibited co-localization signals (Supplementary Figure S6D and E).

Our results showed that Acrs from *Streptococcus* MGEs employed diverse inhibitory strategies to disable Cas9 orthologs in human cells. AcrIIA25.1, AcrIIA26, AcrIIA27, AcrIIA30, AcrIIA31 and AcrIIA32.1 can potently block DNA binding by Cas9 protein. However, AcrIIA24 does not prevent DNA target binding by Cas9 protein, suggesting that AcrIIA24 may specifically inhibit DNA target cleavage by Cas9 with the mechanism similar to AcrIIC1.

### Acrs exhibit diverse mechanisms to inhibit Cas9

We performed EMSAs *in vitro* to further examine the mechanisms of these Acrs against Cas9. RNA EMSAs were conducted to determine whether these Acrs were able to prevent the formation of Cas9–sgRNA RNP complex. We found that AcrIIA25.1, AcrIIA26, AcrIIA27, AcrIIA28 and AcrIIA32.1 had no effect on SpyCas9–sgRNA RNP formation (Supplementary Figure S7A and B). AcrIIA24, AcrIIA30 and AcrIIA31 also did not prevent St3Cas9 or St1Cas9 binding to sgRNA (Supplementary Figure S7C and D). Interestingly, a supershifted band of RNA was observed after the addition of AcrIIA30, either before or after the addition of sgRNA (Supplementary Figure S7D). We speculated that AcrIIA30 may trigger Cas9 RNP complex dimerization to affect Cas9 function, similar to the mechanisms of AcrIIA6 (31) or AcrIIC3 (42).

**Figure 6.**
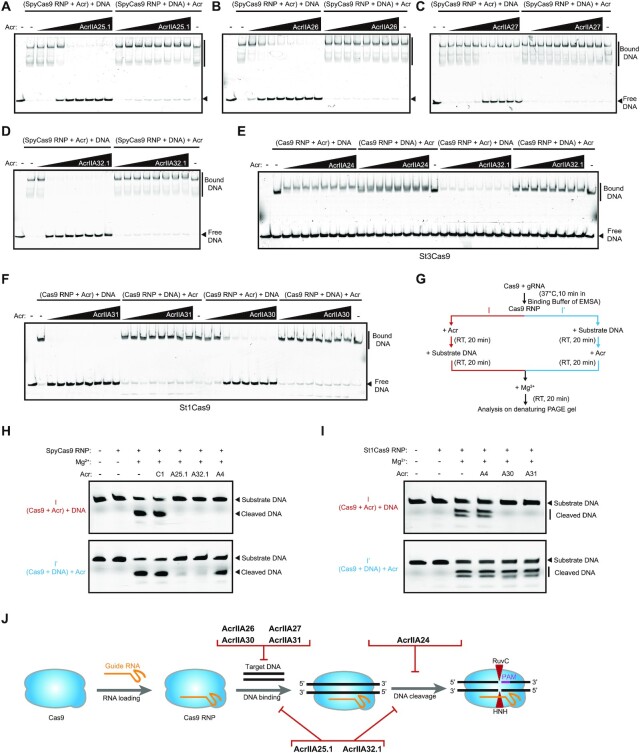
Acrs exhibit diverse mechanisms to inhibit type II-A Cas9 orthologs *in vitro*. EMSAs were conducted to analyze the effect of different Acr proteins on DNA binding of Cas9 RNP, when Acrs were added prior to or after the addition of target DNA, including AcrIIA25.1 (**A**), AcrIIA26 (**B**), AcrIIA27 (**C**), AcrIIA32.1 (**D**), AcrIIA24 (**E**), AcrIIA30 and AcrIIA31 (**F**). Assays were conducted with Cas9 RNP (256 nM) and Acr titrations (0.125, 0.25, 0.5, 0.1, 0.2, 0.4, 0.8 and 1.6 μM). The assays were analyzed on the nondenaturing gel with target DNA labeled by Cy5. The gels are representative of three independent replicates. (**G**) DNA cleavage assays to recover DNA cleavage activity of Cas9 through adding extra Mg^2+^ in EMSAs. The assays were used to examine whether Acrs affect the DNA cleavage activity of Cas9, when added prior to or after the addition of target DNA (see the ‘Materials and Methods’ section for details). RT, room temperature. (**H**, **I**) DNA cleavage assays were conducted to analyze the effect of Acrs on DNA cleavage activity of Cas9 under different conditions shown in panel (G). Acr subtypes and numbers are indicated. A, AcrIIA; C, AcrIIC. Assays were conducted using SpyCas9 RNP (500 nM), St1Cas9 RNP (500 nM), Acrs (10 μM) and substrate DNA (50 nM) with the nontarget strand labeled by Cy3. Experiments were repeated three times and the representative gel figures were shown. (**J**) Summary of different inhibitory mechanisms of anti-CRISPR proteins identified in this study. AcrIIA26, AcrIIA27, AcrIIA30 and AcrIIA31 block Cas9 binding to DNA, while AcrIIA24 abrogates the DNA cleavage activity of Cas9. Remarkably, AcrIIA25.1 and AcrIIA32.1 can inhibit both DNA binding and DNA cleavage of Cas9.

Subsequently, the DNA EMSAs were conducted to investigate how Acrs act on Cas9 RNPs to impact the downstream function of Cas9 RNPs. Cas9 RNPs mixed with catalytically active Cas9 protein and sgRNA were used together with 10 mM EDTA to abrogate target DNA cleavage in the reaction buffer. A fluorescently labeled substrate DNA (Cy5-labeled target strand) probe was designed and targeted by SpyCas9, St1Cas9 and St3Cas9 (Supplementary Table S3). We observed that AcrIIA25.1, AcrIIA26, AcrIIA27, AcrIIA32.1 and control AcrIIA4 efficiently abrogated DNA binding of SpyCas9 RNP, only when added prior to the addition of target DNA (Figure 6A–D and Supplementary Figure S8A–C). Surprisingly, we also found that AcrIIA25.1 and AcrIIA32.1 can trap the DNA-bound Cas9 complex to cause DNA ‘supershift’, while AcrIIA26, AcrIIA27 and the control AcrIIA4 cannot (Figure 6A–D and Supplementary Figure S8A).

Next, we investigated the inhibitory mechanism of AcrIIA32.1 against St3Cas9, and a similar result was achieved compared with AcrIIA32.1 against SpyCas9 (Figure 6E). We also observed that AcrIIA24 can trap the Cas9–DNA complex to cause DNA ‘supershift’ when added before or after the addition of target DNA, while it had no impact on DNA binding of Cas9 RNP (Figure 6E). This result indicated that AcrIIA24 should specifically inhibit DNA target cleavage by St3Cas9. We also examined the inhibitory mechanisms of AcrIIA30 and AcrIIA31 against St1Cas9. We found that both AcrIIA30 and AcrIIA31 efficiently block St1Cas9 RNP binding to DNA, only when added prior to the addition of target DNA (Figure 6F and Supplementary Figure S8D). Like AcrIIA25.1 and AcrIIA32.1, AcrIIA30 can bind to the DNA-bound Cas9 complex to cause DNA ‘supershift’, while AcrIIA31 cannot (Figure 6F and Supplementary Figure S8D).

Considering some unconventional behaviors of AcrIIA25.1, AcrIIA32.1 and AcrIIA30, we speculated that these Acr proteins can not only block DNA binding of Cas9, but also exploit an extra function to inhibit DNA cleavage activity of Cas9 in the RNP–DNA–Acr quaternary complex. To test our hypothesis, we performed DNA cleavage assays by recovering the DNA cleavage activity of Cas9 through adding extra Mg^2+^ in EMSAs (Figure 6G). We examined whether Acr proteins affect the DNA cleavage activity of Cas9, when Acrs were added before or after the addition of target DNA. Our data showed that DNA cleavage activities of SpyCas9, St1Cas9 and St3Cas9 were all effectively recovered by adding extra Mg^2+^ in EMSAs (Figure 6H and I, and Supplementary Figure S8E). Compared to AcrIIC1 protein, AcrIIA25.1, AcrIIA32.1 and control AcrIIA4 can strongly inhibit target DNA cleavage activity of SpyCas9, when added prior to the addition of target DNA. Both AcrIIA25.1 and AcrIIA32.1 can also inactivate the DNA cleavage activity of SpyCas9, when added after the addition of target DNA, in contrast to AcrIIC1 and AcrIIA4 controls (Figure 6H). Combining EMSAs and DNA cleavage assays, our data showed that AcrIIA25.1 and AcrIIA32.1 can inhibit both DNA binding and DNA cleavage activity of SpyCas9, exhibiting unique anti-CRISPR characteristics (Figure 6J).

We further investigated the inhibition of AcrIIA24, AcrIIA25 and AcrIIA32.1 on the DNA cleavage activity of St3Cas9. We found that AcrIIA24, AcrIIA25 and AcrIIA32.1 exhibited robust inhibitions on DNA cleavage of St3Cas9, when added before or after the addition of target DNA (Supplementary Figure S8E). Our results indicated that AcrIIA24 can potently inhibit DNA cleavage step by St3Cas9, while AcrIIA25 and AcrIIA32.1 inhibit both DNA binding and DNA cleavage activity of St3Cas9. We also examined the inhibitory mechanisms of AcrIIA30 and AcrIIA31 on the DNA cleavage activity of St1Cas9, considering that AcrIIA30 can bind to the DNA-bound Cas9 complex in EMSAs. We found that AcrIIA30 and AcrIIA31 can potently inhibit St1Cas9-mediated DNA cleavage, only when added prior to the addition of target DNA, indicating that both AcrIIA30 and AcrIIA31 can inhibit DNA binding of St1Cas9 without impacting DNA cleavage activity of St1Cas9 (Figure 6I and J). Taken together, our results showed that these Acrs we discovered exhibit versatile abilities to inhibit Cas9, with mechanisms including the blockage of DNA binding, DNA cleavage or both.

### Chemically inducible anti-CRISPR variants for the control of CRISPR–Cas9-mediated genome editing

**Figure 7.**
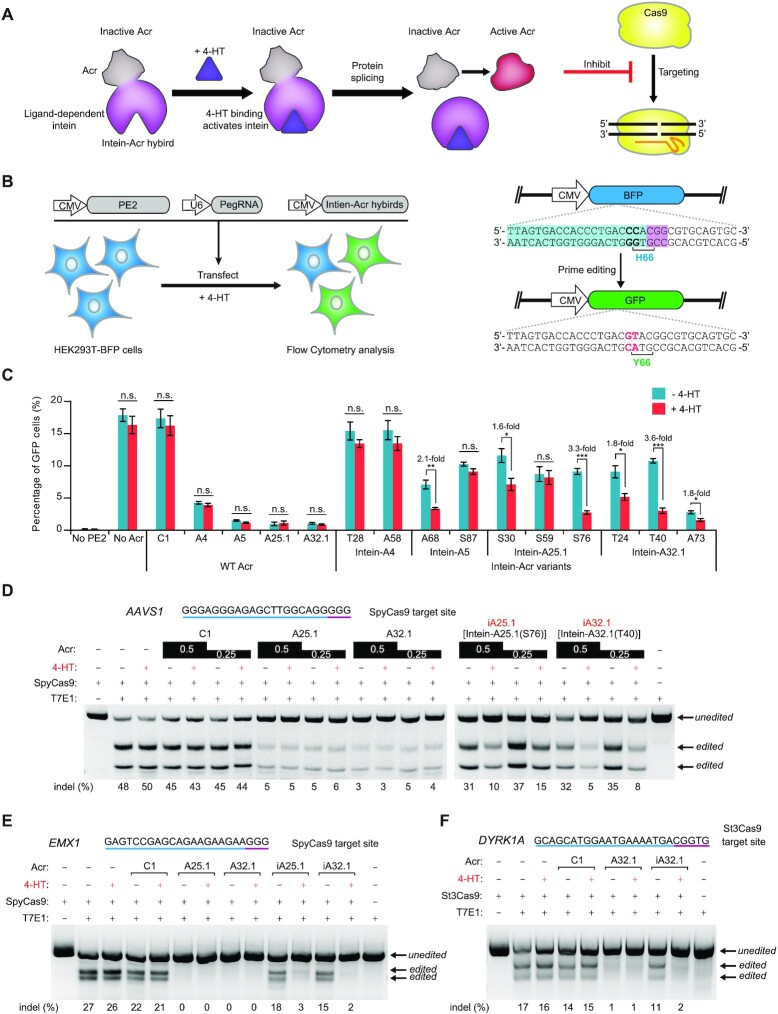
Chemically inducible anti-CRISPR systems for the control of CRISPR–Cas9-mediated genome editing. (**A**) A schematic view of iAcr systems. Insertion of a ligand-dependent intein into Acr protein renders Acr inactive. 4-HT binding can trigger intein protein splicing and restore Acr activity to inhibit Cas9. (**B**) Schematic view of the BFP-to-GFP reporter system for PE to examine the activity of intein–Acr hybrids against Cas9 in human cells. HEK293T cells with a chromosomally integrated BFP (HEK293T-BFP cells) were transfected with plasmids encoding prime editor, BFP-targeting pegRNA and intein–Acr hybrids in the presence or absence of 4-HT (1 μM). The percentage of GFP-positive cells was calculated via flow cytometry at 72 h post-transfection. The PE can switch BFP to GFP by replacing CC to GT, causing single H66Y amino acid substitution. The target sequence, PAM and replaced base are shown in blue, purple and red, respectively. (**C**) Comparison of BFP-to-GFP conversion efficiencies in the presence or absence of wild-type (WT) Acr or intein–Acr variants under the condition of 4-HT treatment or not. Intein–Acr variants are identified by the residue replaced by the intein. WT Acrs including C1 (AcrIIC1), A4 (AcrIIA4), A5 (AcrIIA5), A25.1 (AcrIIA25.1) and A32.1 (AcrIIA32.1) are used as controls. Error bars represent the mean ± SEM with three biological replicates. (**D**) Representative gel images of T7E1 assay to manifest the inhibitory activities of Acr and iAcr proteins against SpyCas9 in the presence or absence of 4-HT. HEK293T cells were transfected with Cas9 (1 μg), sgRNA (0.5 μg) and Acr (0.5 or 0.25 μg) plasmids (see the ‘Materials and Methods’ section for details). The editing efficiencies [indel (%)] are labeled at the bottom of each lane. The target sequence and PAM are highlighted in blue and purple, respectively. The gels are representative of three independent replicates. Representative gel images of T7E1 assay to investigate the inhibitory activities of Acr and iAcr proteins against SpyCas9 (**E**) or St3Cas9 (**F**) in the presence or absence of 4-HT. HEK293T cells were transfected with Cas9 (1 μg), sgRNA (0.5 μg) and Acr (0.25 μg) plasmids (see the ‘Materials and Methods’ section for details). The editing efficiencies [indel (%)] are labeled at the bottom of each lane. The target sites of human *EMX1* (targeted by SpyCas9) and *DYRK1A* (targeted by St3Cas9) are shown at the top of each gel and PAMs are highlighted in purple. The gels are representative of three independent replicates.

Since AcrIIA25.1 and AcrIIA32.1 can inhibit both DNA binding and DNA cleavage activity of Cas9, which manifest powerful potential for modulating Cas9-based applications in genome editing, we selected these two proteins as candidates for developing chemically inducible anti-CRISPRs. We also used AcrIIA4 and AcrIIA5 as controls. We then designed intein–Acr hybrids by Acr protein fusing with a ligand-dependent intein 37R3-2 as previously described (40). Insertion of intein into Acr protein renders Acr inactive, while 4-HT binding to intein can trigger intein protein self-splicing and restore Acr activity to inhibit Cas9 (Figure 7A).

We inserted 4-HT-responsive intein into Acrs by replacing a single residue (cysteine, alanine, serine or threonine) of Acrs, because intein protein splicing leaves behind a single cysteine residue and replacement of these residues will minimize the possible impact of resulting cysteine point mutation (Supplementary Table S4). To examine the effect of intein–Acr hybrids against Cas9 in human cells, we designed a BFP-to-GFP reporter system used for PE (Figure 7B). We established functional HEK293T cells with a chromosomally integrated blue fluorescent protein (HEK293T-BFP cells) at *AAVS1* locus, and flow cytometry analysis revealed high ratio of expression of BFP in HEK293T-BFP cells (Supplementary Figure S9A and B). The PE can switch BFP into GFP by replacing CC to GT, causing single H66Y amino acid substitution (Figure 7B and Supplementary Figure S9C). HEK293T-BFP cells were transfected with plasmids encoding prime editor, BFP-targeting pegRNA and intein–Acr hybrids in the presence or absence of 4-HT (1 μM) (Figure 7B). The effect of intein–Acr hybrids against Cas9 can be calculated by comparing BFP-to-GFP editing efficiencies under each condition.

Our results showed that 4-HT treatment had no obvious effect on the activities of PE and WT Acrs in human cells (Figure 7C). Among 10 intein–Acr variants, the activities of AcrIIA4(T28 and A58), AcrIIA5(S87) and AcrIIA25.1(S59) cannot be regulated by 4-HT. Although AcrIIA5(A68), AcrIIA25.1(S30) and AcrIIA32.1(T24 and A73) can switch to active state to inhibit Cas9 through responses to 4-HT treatment, these four intein–Acr variants were weakly 4-HT dependent (average 1.8-fold regulation). Only two intein–Acr variants, AcrIIA25.1(S76) and AcrIIA32.1(T40), can inhibit PE-mediated BFP-to-GFP editing in the presence of 4-HT, exhibiting 4-HT-dependent regulation (with 3.3- and 3.6-fold changes, respectively). We then referred to intein–AcrIIA25.1(S76) and intein–AcrIIA32.1(T40) as iAcrIIA25.1 (abbreviated as iA25.1) and iAcrIIA32.1 (abbreviated as iA32.1), respectively.

To further examine the 4-HT-dependent activities of iA25.1 and iA32.1 to inhibit Cas9-mediated gene editing, we performed T7E1 assays in HEK293T cells. Human endogenous *AAVS1* and *EMX1* loci were designed for gene editing by SpyCas9. Consistent with the results from PE-mediated BFP-to-GFP assays, 4-HT treatment had no effect on the activities of SpyCas9 and WT Acrs (Figure 7D and E). Strikingly, we observed that the inhibitory activities of iA25.1 and iA32.1 were 4-HT dependent. These two proteins have slight effect on SpyCas9 activity without 4-HT, but they switch to active state to inhibit Cas9 in the presence of 4-HT (Figure 7D and E). To further examine the potential applications of iAcr, we conducted T7E1 assays to investigate whether iA32.1 can be activated by 4-HT to inhibit St3Cas9-mediated gene editing in human cells or not, given that AcrIIA32.1 is able to block St3Cas9 activity as well. As expected, our data showed that iA32.1 with 4-HT-triggered activity can suppress St3Cas9-mediated gene editing in human cells (Figure 7F). To assess the 4-HT-dependent activities of iA25.1 and iA32.1 to inhibit Cas9-mediated gene editing, we also performed NGS on *EMX1* (targeted by SpyCas9) and *DYRK1A* (targeted by St3Cas9) loci. Consistent with the results of T7E1 assays, we found that the inhibitory activities of iA25.1 and iA32.1 were indeed 4-HT dependent (∼5–7-fold change) (Supplementary Figure S10A and B). Thus, our results showed these iAcrs exhibit 4-HT-dependent regulation to post-translationally control CRISPR–Cas9-mediated genome editing in human cells.

## DISCUSSION

In response to long-term selective pressures, MGEs evolved diverse anti-CRISPR proteins to combat CRISPR–Cas adaptive immune systems in prokaryotes (11). To date, 88 distinct Acr families have been discovered to inhibit multiple types of CRISPR–Cas systems, indicating their important roles in CRISPR–Cas biology and phage–host interactions (15). Here, we identified nine distinct *anti-CRISPR* genes (*AcrIIA24–32*) in *Streptococcus* MGEs, using the ‘guilt-by-association’ bioinformatics approach and screening system in *E. coli*. AcrIIA24–32 are specific inhibitors of the *Streptococcus* CRISPR–Cas9 systems, which can inhibit type II-A Cas9 orthologs from *Streptococcus* (SpyCas9, St1Cas9 and St3Cas9). Through comprehensive phylogenetic analyses, we also found that *AcrIIA24–32* families are mainly distributed in *Streptococcus*. These results suggest that Acr proteins may play an important role in the evolutionary arms race between phages and hosts in *Streptococcus*.

All known Acr proteins typically exhibit low molecular weight and lack conserved sequence and structural features, which make it difficult to predict *de novo* Acr proteins and their functions. Aca protein usually contains a DNA-binding domain such as HTH for regulation of *acr* gene expression, which can be used as markers for the discovery of novel Acr proteins using the guilt-by-association approach (33,51,52). In our study, we have identified three new *aca* genes (*aca11–13*) in *Streptococcus* MGEs, which can further facilitate the discovery and characterization of relevant Acr proteins. We also found that the N- or C-terminal portion of some Acrs and their orthologs (i.e. AcrIIA24, AcrIIA29, AcrIIA31 and AcrIIA32 families) fuses with Aca protein, similar to AcrIIA13–15 (23). Because of the similar inhibitory activity between AcrIIA32 (fusion with Aca13) and AcrIIA32.1 (without fusion with any Aca protein) in bacteria, human cells and even *in vitro*, we speculate that the Aca region with fusion motif in these Acr proteins should have no obvious impact on Acr potency. Although the possible self-regulation role of Aca should be further elucidated, this Aca and Acr fusion feature may be used as a more confident marker for the discovery of new Acr proteins.

All elucidated Acr proteins have been shown to inhibit the CRISPR interference stage, including crRNA loading interference, DNA binding prevention and DNA cleavage blockage (24,28,36). In this study, the Acrs we discovered exhibit versatile abilities to inhibit Cas9 with diverse mechanisms. Among these Acr proteins, AcrIIA24 abrogates the DNA cleavage activity of Cas9, suggesting that AcrIIA24 may target the nuclease domain of Cas9, which is similar to the mechanisms of AcrIIC1 (42). We also found that AcrIIA26, AcrIIA27, AcrIIA30 and AcrIIA31 are able to block Cas9 binding to DNA, which is reminiscent of AcrIIA4 and AcrIIA6 (28,31). Remarkably, AcrIIA25.1 and AcrIIA32.1 inhibit both DNA binding and DNA cleavage activity of Cas9, exhibiting unique anti-CRISPR characteristics. This unique mechanism of AcrIIA25.1 and AcrIIA32.1 may allow phages to escape more effectively from CRISPR interference, and reduce phage multiplicity of infection threshold, which is a critical parameter for infection of Acr-carrying phage as previously described (53). Future work on the detailed mechanisms of AcrIIA24–32 with structural or molecular information will be necessary to understand how these proteins interact with Cas9 in detail. In addition, the dual functional capability of AcrIIA25.1 and AcrIIA32.1 gives us new insights into AcrIIA5, given that the inhibitory mechanism of AcrIIA5 against Cas9 has been debated in recent publications (36,54,55). In our previous study and this study, AcrIIA5 specifically prevents DNA cleavage activity of Cas9 *in vitro* and *in vivo*, while other reports indicated that AcrIIA5 can inhibit target DNA binding of Cas9 *in vivo* (54,56). Considering that these previous studies assessed AcrIIA5 function based only on indirect measurements, this discrepancy may be due to the effects of AcrIIA5 in changing protein expression profiles in cells (36). Here, we proposed another hypothesis that AcrIIA5 might be a dual functional protein with different tendencies of affecting target DNA binding and DNA cleavage of Cas9 under different experimental conditions. Thus, the exact mechanism of AcrIIA5 to inhibit Cas9 needs to be further explored.

Chemically inducible strategies for post-translational control of protein activity have proven to be effective methods (40,57), while chemically inducible anti-CRISPR systems used for genome editing in human cells have not been described. Here, we successfully developed chemically inducible anti-CRISPR systems based on AcrIIA25.1 and AcrIIA32.1 for the regulation of Cas9-based genome editing. In our study, both iA25.1 and iA32.1 display 4-HT-dependent regulation for post-translational control of CRISPR–Cas9-mediated genome editing in human cells. Compared to other strategies for regulating Cas9 activity, iAcr systems have several unique features that make them good candidates for certain applications. (i) In our work, we observed that iA32.1 with 4-HT-triggered activity can not only modulate SpyCas9 and St3Cas9-mediated gene editing, but also regulate PE in human cells. Therefore, iAcr strategy may achieve broad-spectrum regulation of multiple CRISPR–Cas9 systems and Cas9 effector systems. Additionally, current iAcr strategy minimizes the need of cumbersomely engineering each Cas9 nuclease or finding small molecules to inhibit them (12,58–60). (ii) Due to small sizes of iA25.1 (57.8 kDa) and iA32.1 (57.2 kDa), these iAcrs can easily be carried by plasmids or viral vectors to obtain efficient Cas9 modulation *in vitro* and *in vivo*. (iii) These iAcrs provide the ‘off-switch’ systems for modulating Cas9 activity, which are complementary to other existing strategies, especially for those ‘on-switch’ systems (61,62). Additionally, some type II Acr proteins have been developed to limit off-target effects by CRISPR–Cas9 under laboratory conditions (44,63,64). These iAcrs can modulate Cas9-mediated gene editing with potentiality to improve the specificity of Cas9, which needs to be further investigated. Overall, iAcr strategy in our work exhibits promising applications in the future and is a starting point to develop chemically inducible anti-CRISPR systems.

We are also curious about the origin and evolution of Acr proteins, which is still unclear. A possible hypothesis is that Acr proteins may derive from the structural protein of bacteriophages, considering that peptides derived from the periplasmic domain of phage major coat protein G8P (G8P_PD_) of Inoviridae bacteriophages can inhibit SpyCas9 (31).

In summary, we discovered multiple CRISPR–Cas9 inhibitors from *Streptococcus* MGEs and these Acrs exhibited diverse mechanisms to inhibit Cas9. We also developed chemically inducible anti-CRISPR systems (iA25.1 and iA32.1), which exhibit 4-HT-dependent regulation to post-translationally control CRISPR–Cas9-mediated genome editing in human cells. Our work expands the diversity of type II-A anti-CRISPR families and their inhibitory mechanisms, providing novel insights into the evolutionary arms race between phages and hosts and strategies for developing Acr-associated controllable gene editing tools.

## DATA AVAILABILITY

All data generated during this study are included in this article. Additional DNA sequences, amino acid sequences and accession numbers of AcrIIA and Aca proteins are provided in Supplementary Table S1.

## Supplementary Material

gkac099_Supplemental_Files
