# Supplementary material for: Discovery of potent and versatile CRISPR–Cas9 inhibitors engineered for chemically controllable genome editing
Source: Nucleic Acids Res. 2022 Feb 21;50(5):2836–53. doi: 10.1093/nar/gkac099 (PMC8934645; doi:10.1093/nar/gkac099)
Supplement: gkac099_Supplemental_Files [file gkac099_supplemental_files.zip › Supplementary Figures.pdf]

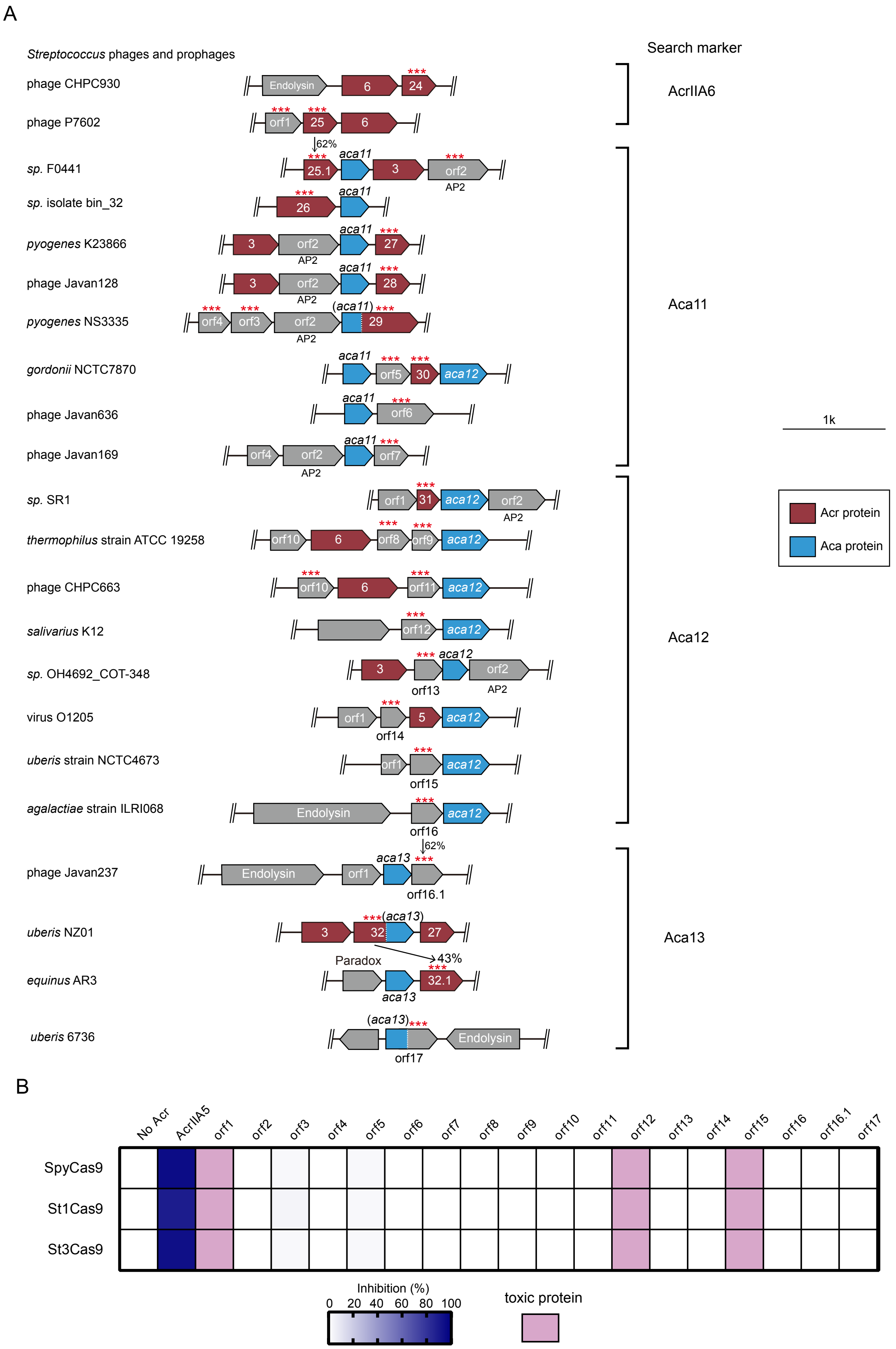

Supplementary Figure S1. Schematic view and screening of candidate *acr* genes. (**A**) Full schematic view of candidate *acr*, *aca*, and relevant neighbouring genes in the genome of *Streptococcus* phages and prophages, related to Figure 1A. *Acr* genes are shown in red with numbers. Arrows indicate the relationship between *acr* loci

with the percentage of protein sequence identity. *Aca* genes are shown in blue with numbers. Other neighboring genes are shown in gray and some known genes are annotated according to the NCBI website. AP2 DNA binding motifs were detected by HHpred (see the 'Materials and Methods' section). Individual genes (***) were assayed for CRISPR-Cas9 inhibition in *E. coli*, open reading frame (orf). (**B**) A matrix showing the inhibitory activity of other candidate *Acr* genes (*orf1-17*) against type II-A Cas9 orthologs. Samples labeled with light red represent toxic proteins expressed in *E. coli* (i.e. no clones exist in plasmid-interference assays with *E. coli* transformed with mismatching spacer Cas9 plasmids). Values of this figure represent the mean of at least three biological replicates.

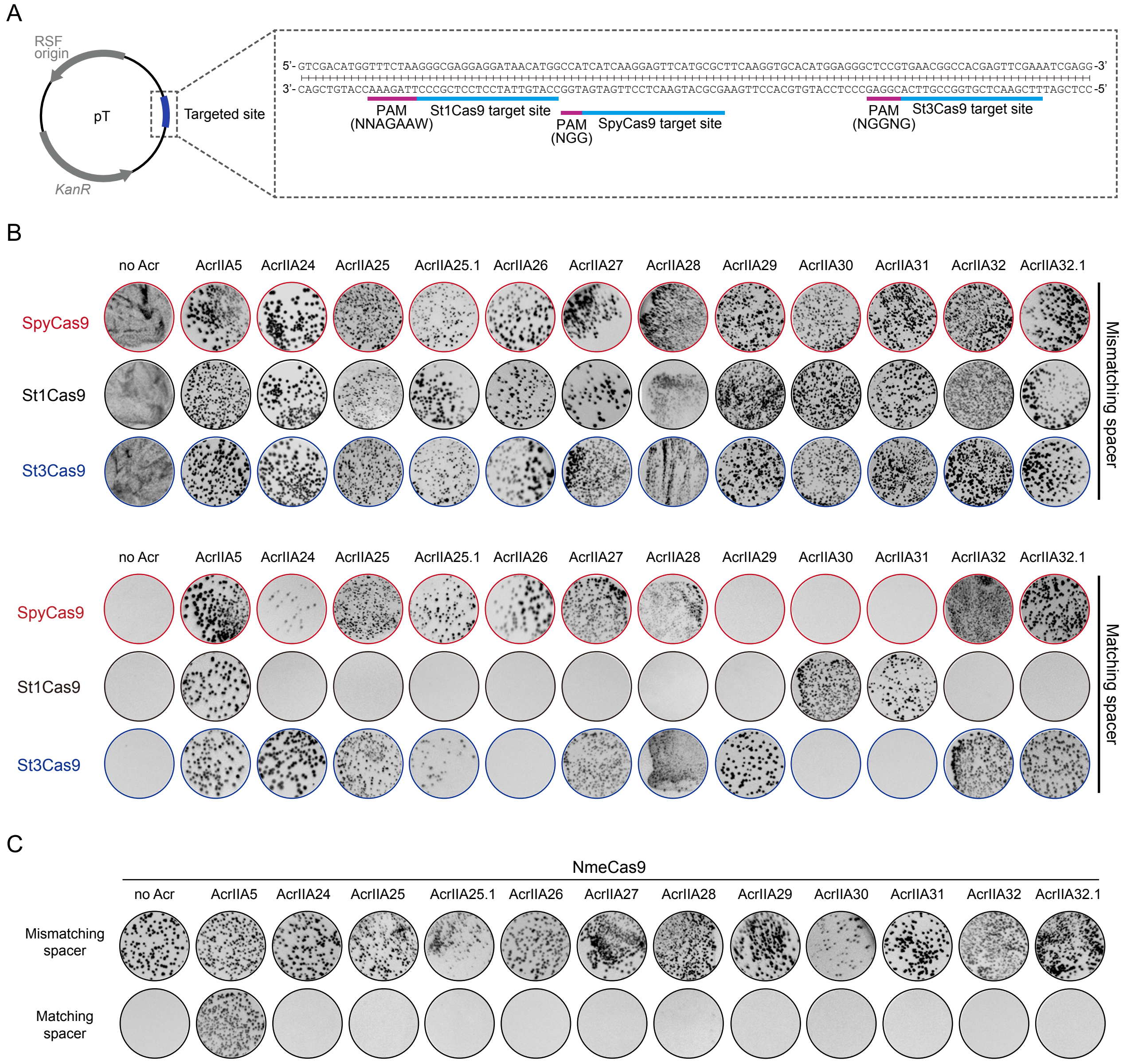

Supplementary Figure S2. Acr candidates tested for inhibitory activities against type II-A Cas9 orthologs in *E. coli*. (**A**) Schematic representation of pT containing target sites of Cas9 orthologs with matching spacer and respective PAMs. (**B**) Representative colony pictures of plasmid-interference assays in *E. coli*, showing the inhibitory activities of AcrIIA24-32 against type II-A Cas9 orthologs (SpyCas9, St1Cas9, and St3Cas9). *E. coli* carrying Acr plasmids were co-transformed with pT and Cas9 (mismatching or matching spacer) plasmids, related to Figure 1C. (**C**) Representative colony pictures of plasmid-interference assays to investigate the inhibitory activities of AcrIIA24-32 against NmeCas9.

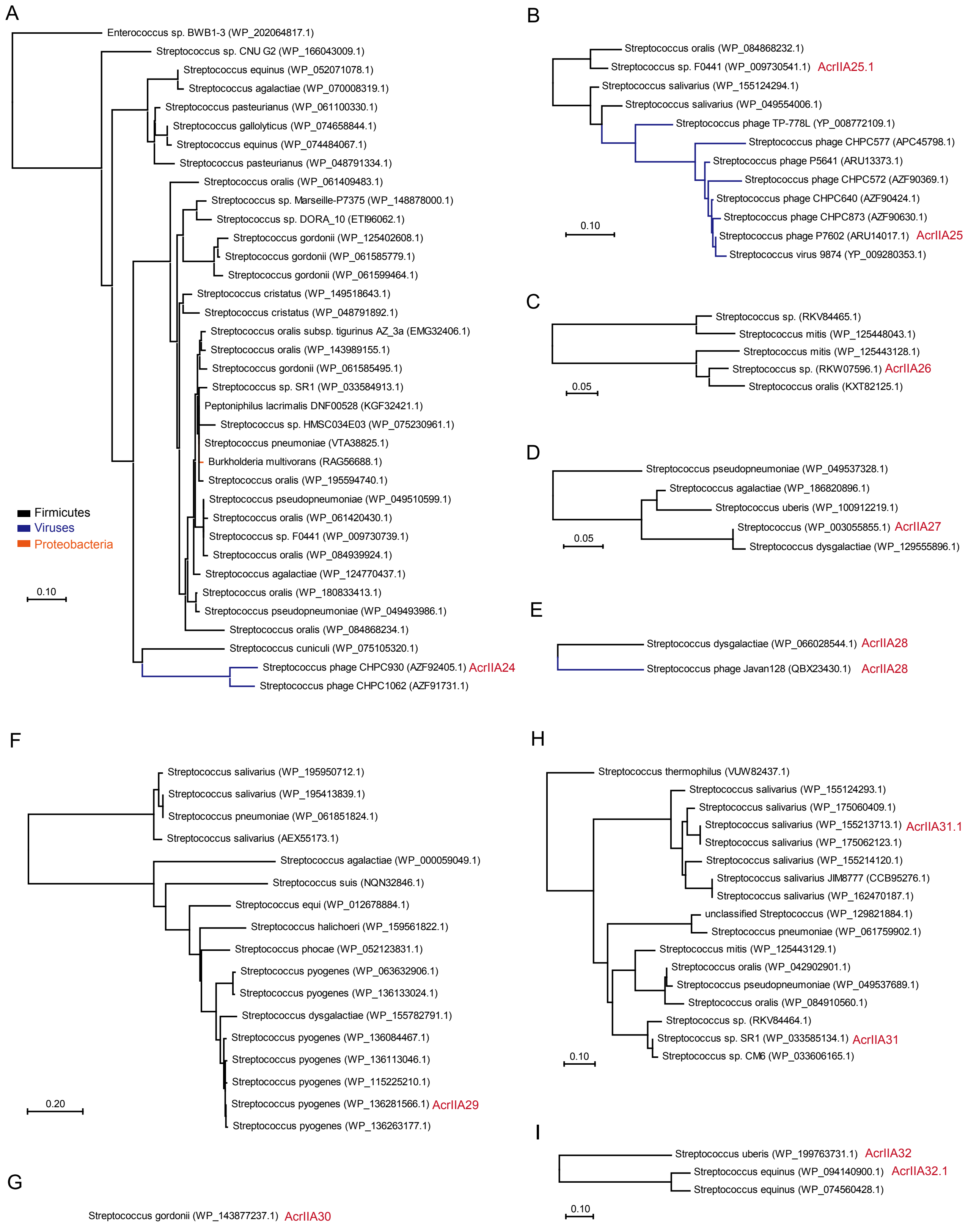

Supplementary Figure S3. Phylogenetic analysis of AcrIIA24-32 orthologs. (**A-I**) Minimum evolution phylogenetic tree of AcrIIA24-32 orthologs (panels **A** to **I**, respectively) with protein sequences identified through BLASTp search. Acr proteins analyzed in this study are marked behind the species in red color.

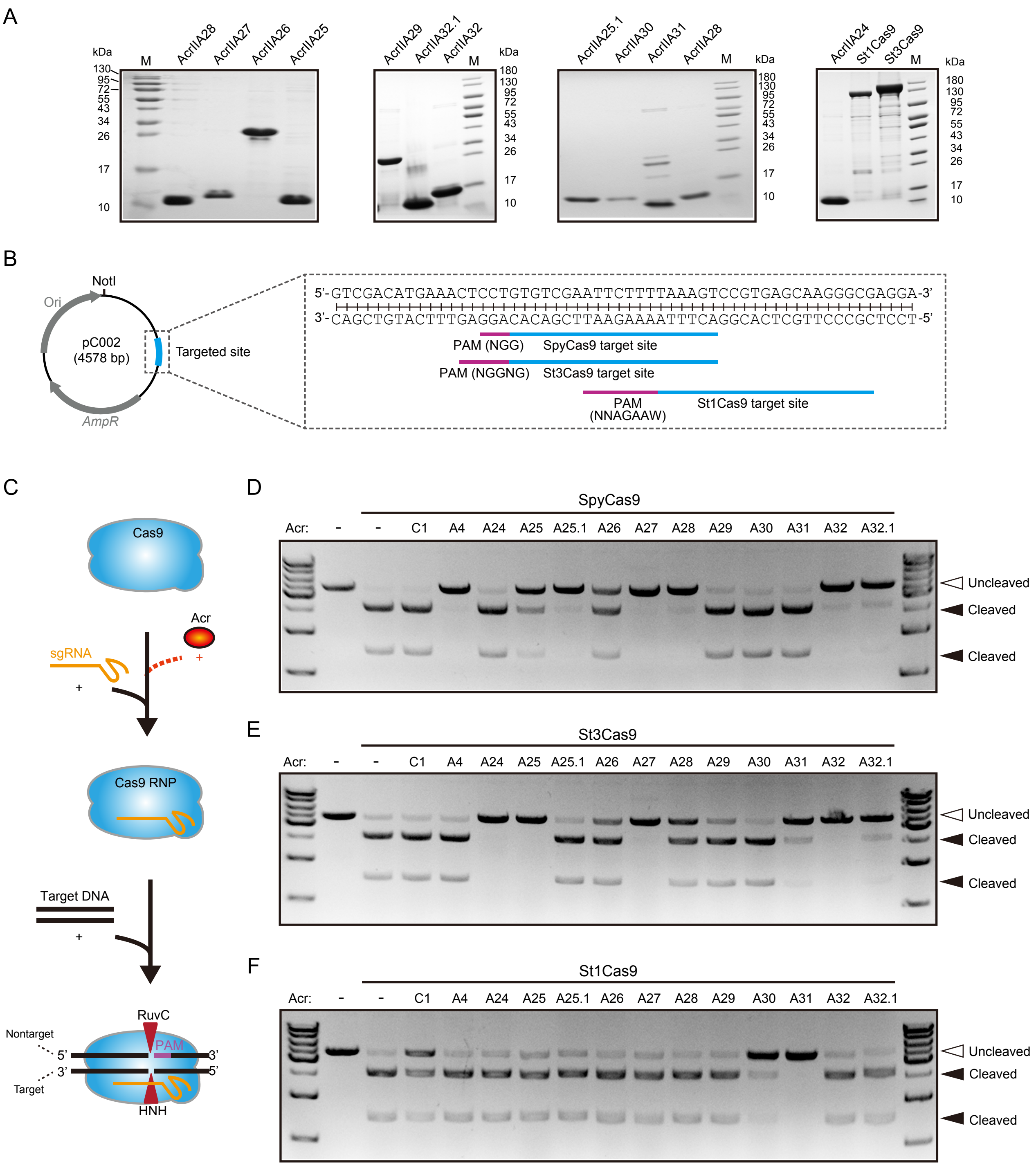

Supplementary Figure S4. Purification of Cas9 and Acr proteins and analysis of Acr inhibitory activities *in vitro*. (**A**) SDS-PAGE gel images show the purified St1Cas9, St3Cas9, and Acr proteins used in this study. St1Cas9 is from *Streptococcus thermophilus* LMD-9 CRISPR1, and St3Cas9 is from *Streptococcus thermophilus* LMD-9 CRISPR3. See Supplementary Table S1 and S5 for protein sequence information. (**B**) Schematic representation of the target DNA sites of different Cas9 orthologs and respective PAMs used in DNA cleavage assay. Target DNA was generated by linearizing pC002 plasmid through NotI restriction endonuclease. (**C**) Procedure of DNA cleavage assays under different conditions corresponding to Figure 3A. DNA cleavage assays using pre-incubated apo-Cas9 with Acr proteins before sgRNA and target DNA were introduced into the reaction. (**D-F**) DNA cleavage assays targeting linearized plasmid DNA by SpyCas9 (**D**), St3Cas9 (**E**), and St1Cas9 (**F**) in the presence or absence of Acr proteins. Acr subtypes and numbers are indicated. A, AcrIIA; C, AcrIIC. Hollow arrowheads indicate the uncleaved linearized plasmid DNA. Solid arrowheads indicate the cleaved products from linearized plasmid DNA. The DNA cleavage assays shown are representative of three independent replicates.

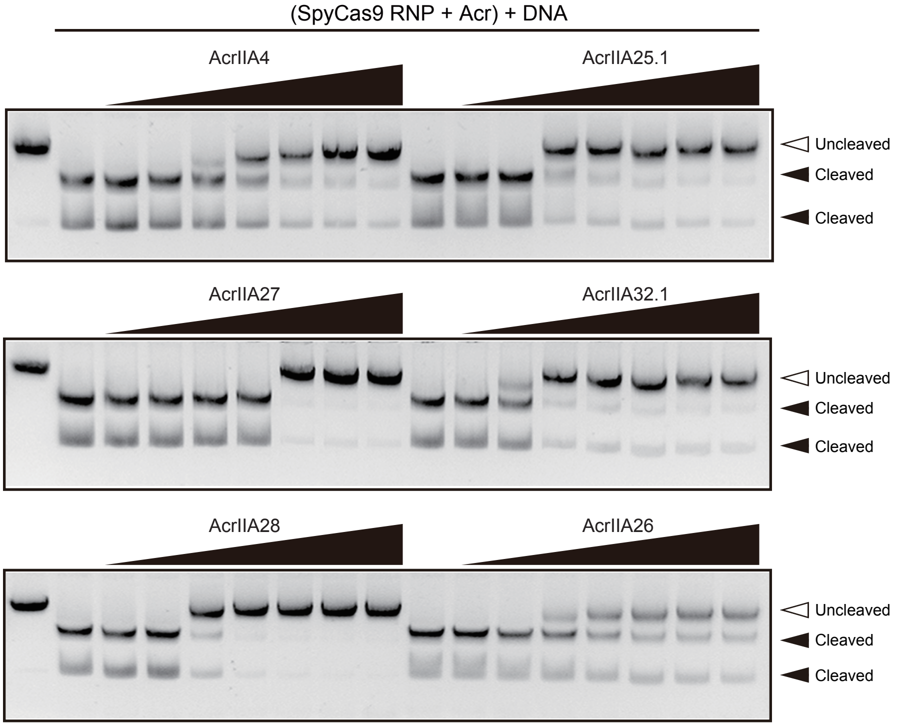

Supplementary Figure S5. Dose-dependent inhibitory activities of Acrs against Cas9 proteins *in vitro*. DNA cleavage assays were conducted to analyze the dose-dependent effect of Acr proteins on SpyCas9. Assays were conducted with SpyCas9 RNP (256 nM) and Acr titrations (0, 128, 256, 512, 1024, 2048, 4096 and 8192 nM). Hollow arrowheads indicate the uncleaved linearized plasmid DNA. Solid arrowheads indicate the cleaved products from linearized plasmid DNA. The DNA cleavage assays shown are representative of three independent replicates.

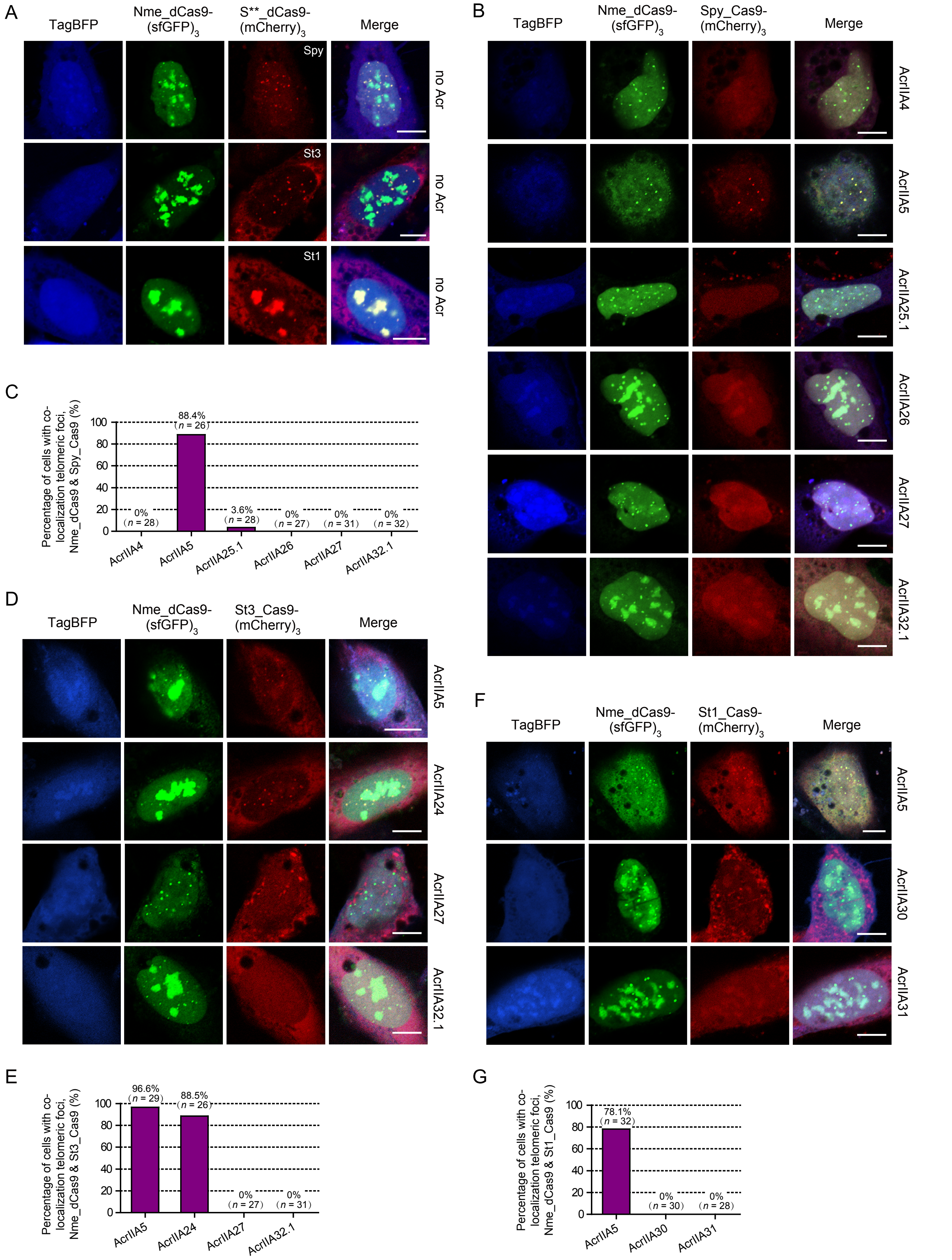

Supplementary Figure S6. Restoring catalytic activities of type II-A dCas9 orthologs in fluorescence localization assays for human telomeric foci. (**A**) Representative images of U2OS cells after transfection with Nme_dCas9-$(sfGFP)_3$ and Acr empty (no Acr) plasmids, along with Spy_dCas9-$(mCherry)_3$, St1_dCas9-$(mCherry)_3$, or

St3_dCas9-(mCherry)$_3$ plasmids. The scale bars represent 10 μm. (**B-G**) Representative images of U2OS cells after transfection with Nme_dCas9-(sfGFP)$_3$, Spy_Cas9-(mCherry)$_3$ (**B**), St3_Cas9-(mCherry)$_3$ (**D**) or St1_Cas9-(mCherry)$_3$ (**F**), along with different Acr plasmids. The fluorescent channels are shown at the top of the figure, and different Acr proteins are shown at the right of each row. The scale bars represent 10 μm. Quantitation of Spy_Cas9-(mCherry)$_3$ (**C**), St3_Cas9-(mCherry)$_3$ (**E**), and St1_Cas9-(mCherry)$_3$ (**G**) telomeric foci under each condition was counted based on the co-localization with Nme_dCas9-(sfGFP)$_3$ in the presence of different Acr proteins. Foci were scored blind (see the 'Materials and Methods' section for details). *n* = number of cells that were scored under each condition.

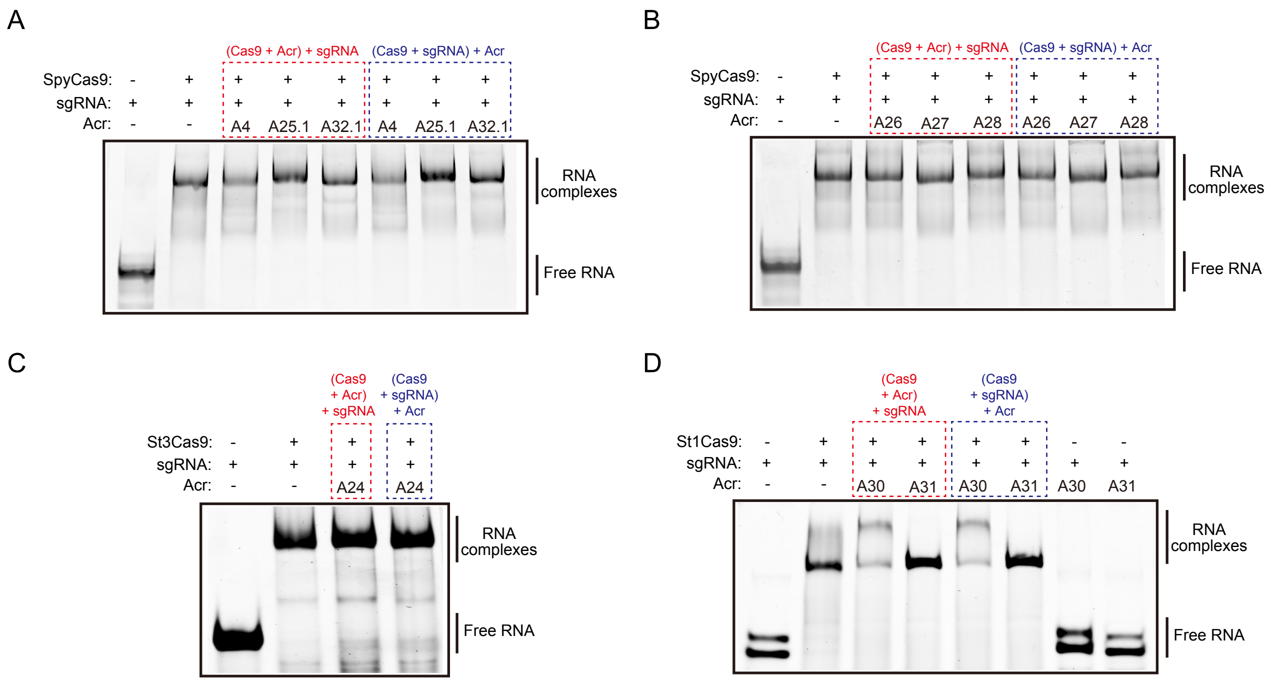

Supplementary Figure S7. Acrs have no effect on the formation of Cas9-sgRNA RNP complex. (**A** and **B**) EMSA analysis of SpyCas9 binding to sgRNA in the presence or absence of Acrs including AcrIIA25.1, AcrIIA26, AcrIIA27, AcrIIA28 and AcrIIA32.1. The assay was analyzed on a native gel with the sgRNA visualized by SYBR gold. Orders of the addition of different reaction components (Acrs, SpyCas9 and sgRNA) are shown above the red or blue boxes. (**C**) EMSA assay was conducted to analyze the effect of AcrIIA24 protein on St3Cas9 binding to sgRNA, when AcrIIA24 was added prior to or after the addition of sgRNA. (**D**) EMSA analysis of St1Cas9 binding to sgRNA in the presence or absence of AcrIIA30 or AcrIIA31.

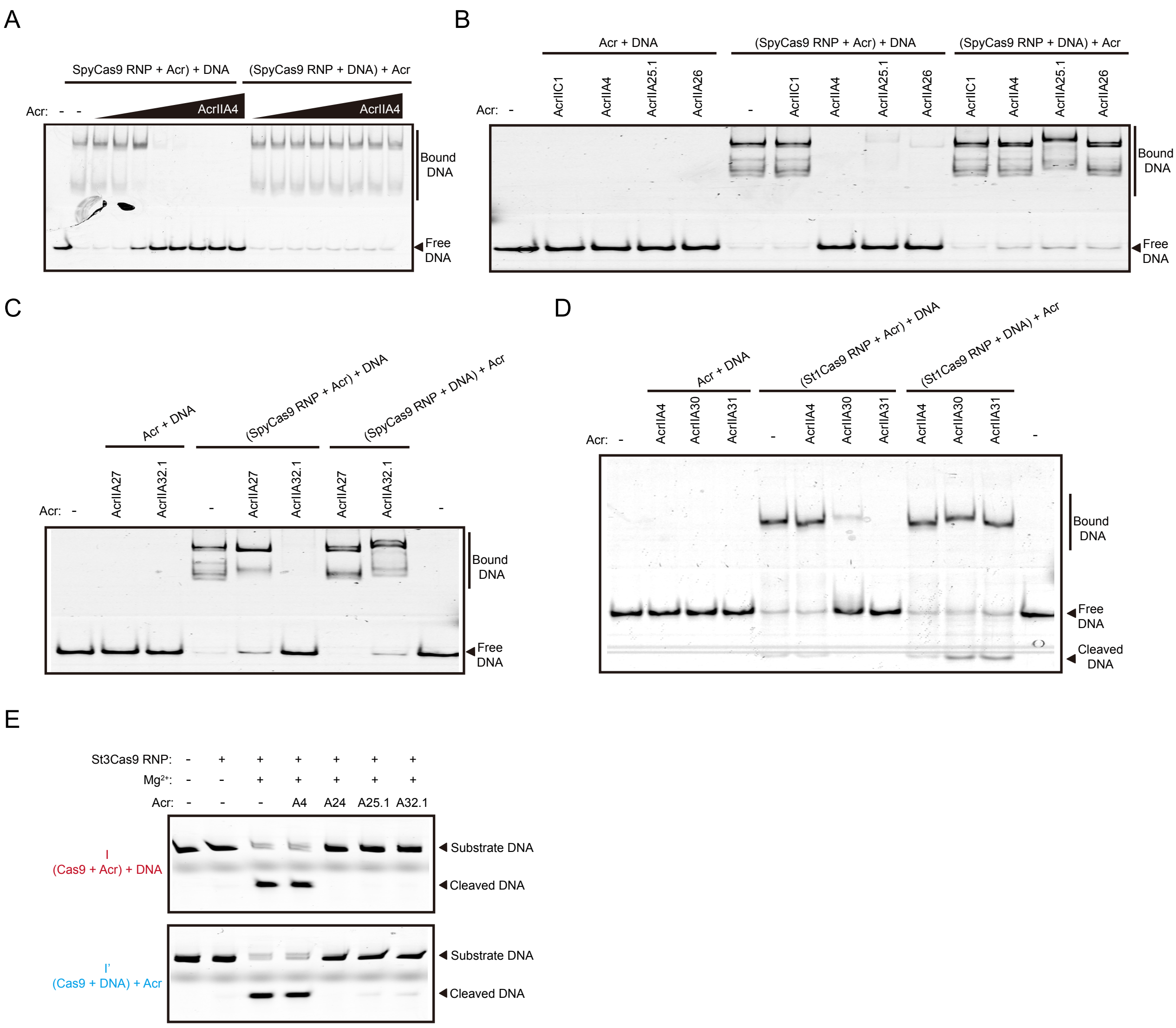

Supplementary Figure S8. Acr proteins inhibit Cas9 through diverse approaches. (**A**) EMSA assays were conducted to analyze the effect of AcrIIA4 on DNA binding of SpyCas9 RNP, when added prior to or after the addition of target DNA. The assays were analyzed on the non-denaturing gel with target DNA labeled by Cy5. Assays were conducted with SpyCas9 RNP (256 nM) and AcrIIA4 titrations (64, 128, 256, 512, 1024, 2048, 4096 and 8192 nM). (**B-D**) EMSA analysis of Cas9 binding to DNA in the presence or absence of different Acrs. The regents (Acrs, Cas9, sgRNA, and substrate DNA) were incubated in different orders. Assays were conducted with SpyCas9 RNP (256 nM), St1Cas9 RNP (256 nM), Acrs (1.6 μM), and substrate DNA (50 nM) using the target strand labeled by Cy5. Experiments were repeated at least three times. (**E**) DNA cleavage assays were conducted to analyze the inhibition of St3Cas9 cleavage activity by AcrIIA24, AcrIIA25, and AcrIIA32.1 under different conditions shown in Figure 6G. Acr subtypes and numbers are indicated. A, AcrIIA. Assays were conducted with St3Cas9 RNP (500 nM), Acrs (10 μM), and substrate DNA (50 nM) using the target strand labeled by Cy5. Experiments were repeated at least three times.

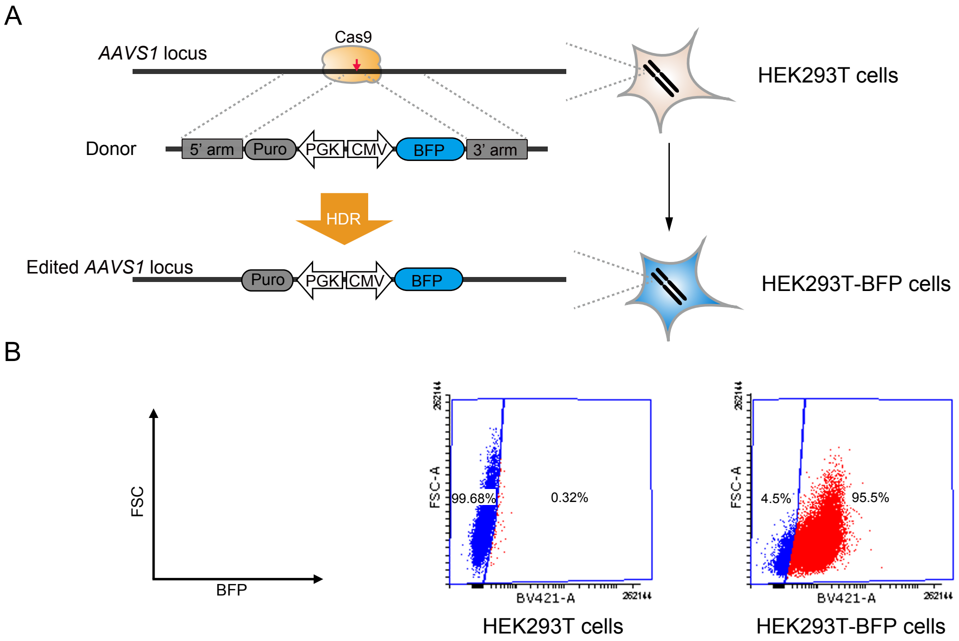

C

PegRNA	Target sequence	Sequence for RT template and PBS	Desired edit	PBS length	RT length	Transcription product of PegRNA (Spacer, RT template, sgRNA scaffold, and PBS)
SpyCas9-PegRNA-BFP	TTAGTGACCACCCTGACCCA**CGG**	TGCACGCCGTACGTCAGGGTGGTCA	+1-2 CC to GT	13	12	UUAGUGACCACCCUGACCCAGUUUUAGAGCUAGAAAUAGCAAGUUAAAAUAAGGCUAGUCCGUUAUCAACUUGAAAAAGUGGCACCGAGUCGGUGCUGCACGCCGUACGUCAGGGUGGUCAUUU

Supplementary Figure S9. Cas9-mediated integration of BFP cassette at the *AAVS1* locus in human HEK293T cells. (**A**) Schematic view of establishing HEK293T-BFP cell lines using Cas9-mediated homology-directed repair (HDR) at the *AAVS1* locus. A donor vector was designed with *BFP* and *puromycin* resistant genes driven by the CMV and PGK promoters, respectively. (**B**) The percentage of BFP-positive cells measured by flow cytometry. (**C**) The design of BFP-targeting pegRNA in BFP-to-GFP reporter system for prime editing.

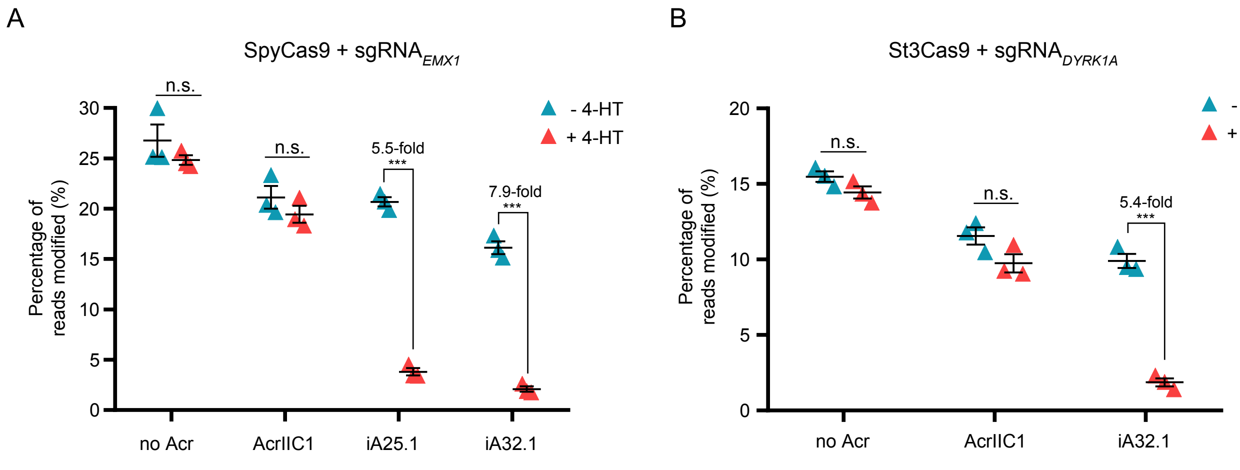

Supplementary Figure S10. Next-generation sequencing analysis of the inhibitory activities of iAcr proteins against Cas9. (**A** and **B**) HEK293T cells were transfected with plasmids targeting the *EMX1* (**A**, by SpyCas9) or *DYRK1A* (**B**, by St3Cas9) locus and Acr or iAcr variants in the presence or absence of 4-HT. Editing efficiencies were assessed by next-generation sequencing and percentage of reads modified are represented as the means ± SEM from three individual experiments.

Supplementary Table S2. Primers and target sequences used in T7E1 and NGS assays

**Cas9**	**Target**	**Target sequences (5'-3') (The PAM sequence is in underlined)**	**T7E1-F (5'-3')**	**T7E1-R (5'-3')**	**NGS-F (5'-3')**	**NGS-R (5'-3')**
SpyCas9	human *AAVS1* locus	GGGACCACCTTATATTCCCA**GGG**	TGCTTTCTTTGCCTGGACAC	CCTCTCTGGCTCCATCGTAA	–	–
SpyCas9	human *AAVS1* locus	GGGAGGGAGAGCTTGGCAGG**GGG**	TGGCTACTGGCCTTATCTCACAGG	CTCTCTAGTCTGTGCTAGCTCTTCCAG	–	–
SpyCas9	human *EMX1* locus	GAGTCCGAGCAGAAGAAGAA**GGG**	GCCCCTAACCCTATGTAGCCTCAG	CATTGCTTGTCCCTCTGTCAATGG	TCGTCGGCAGCGTCAGATGTGTATAAGAGACAGAGGTGAAGGTGTGGTTCCAG	GTCTCGTGGGCTCGGAGATGTGTATAAGAGACAGAGTGGCCAGAGTCCAGCTT
St1Cas9	human *DYRK1A locus*	TGAATCTGGTCAGAATATGATAAG**GCAGAAA**	GGAGCTGGTCTGTTGGAGAA	TCCCAATCCATAATCCCACGTT	–	–
St3Cas9	human *DYRK1A locus*	GCAGCATGGAATGAAAATGA**CGGTG**	GGAGCTGGTCTGTTGGAGAA	TCCCAATCCATAATCCCACGTT	TCGTCGGCAGCGTCAGATGTGTATAAGAGACAGATTTAAGGAACCATTAGATATGTC	GTCTCGTGGGCTCGGAGATGTGTATAAGAGACAGAGGTTGCTGAATCTGGTCAG

Supplementary Table S3. Oligonucleotides of substrate DNA used in this study.

Name	Sequences (5'-3')
Substrate DNA-nontargeting strand	CGACTACTCCTCGCGGGAGCAAGGTCTGCCACTGAGTGGAGAATGACACCCGTGAG
Substrate DNA-nontargeting strand labeled with Cy3 (* represents fluorescein)	*CGACTACTCCTCGCGGGAGCAAGGTCTGCCACTGAGTGGAGAATGACACCCGTGAG
Substrate DNA-targeting strand	CTCACGGGTGTCATTCTCCACTCAGTGGCAGACCTTGCTCCCGCGAGGAGTAGTCG
Substrate DNA-nontargeting strand labeled with Cy5 (* represents fluorescein)	*CTCACGGGTGTCATTCTCCACTCAGTGGCAGACCTTGCTCCCGCGAGGAGTAGTCG

Supplementary Table S4. The amino acid sequences of intein-Acr variants designed in this study.

Name	Amino acid sequences (intein, Acr)
**Intein-AcrIIA4(T28)**	MNINDLIREIKNKDYTVKLSGTDSNSICLAEGTRIFDPVTGTTHRIEDVVDGRKPIHVVAAAKDGTLLARPVVSWFDQGTRDVIGLRIAGGAIVWATPDHKVLTEYGWRAAGELRKGDRVAGPGGSGNSLALSLTADQMVSALLDAEPPILYSEYDPTSPFSEASMMGLLTNLADRELVHMINWAKRVPGFVDLTLHDQAHLLERAWLEILMIGLVWRSMEHPGKLLFAPNLLLDRNQGKCVEGMVEIFDMLLATSSRFRMMNLQGEEFVCLKSIILLNSGVYTFLSSTLKSLEEKDHIHRALDKITDTLIHLMAKAGLTLQQQHQRLAQLLLILSHIRHMSNKGMEHLYSMKYKNVVPLYDLLLEMLDAHRLHAGGSGASRVQAFADALDDKFLHDMLAEGLRYSVIREVLPTRRARTFDLEVEELHTLVAEGVVVHNCQLIIRVNNDGNEYVISESENESIVEKFISAFKNGWNQEYEDEEEFYNDMQTITLKSELN
**Intein-AcrIIA4(A58)**	MNINDLIREIKNKDYTVKLSGTDSNSITQLIIRVNNDGNEYVISESENESIVEKFISCLAEGTRIFDPVTGTTHRIEDVVDGRKPIHVVAAAKDGTLLARPVVSWFDQGTRDVIGLRIAGGAIVWATPDHKVLTEYGWRAAGELRKGDRVAGPGGSGNSLALSLTADQMVSALLDAEPPILYSEYDPTSPFSEASMMGLLTNLADRELVHMINWAKRVPGFVDLTLHDQAHLLERAWLEILMIGLVWRSMEHPGKLLFAPNLLLDRNQGKCVEGMVEIFDMLLATSSRFRMMNLQGEEFVCLKSIILLNSGVYTFLSSTLKSLEEKDHIHRALDKITDTLIHLMAKAGLTLQQQHQRLAQLLLILSHIRHMSNKGMEHLYSMKYKNVVPLYDLLLEMLDAHRLHAGGSGASRVQAFADALDDKFLHDMLAEGLRYSVIREVLPTRRARTFDLEVEELHTLVAEGVVVHNCFKNGWNQEYEDEEEFYNDMQTITLKSELN
**Intein-AcrIIA5(A68)**	MAYGKSRYNSYRKRSFNRSNKQRREYAQEMDRLEKAFENLDGWYLSSMKDSAYKDFGKYEIRLSNHSCLAEGTRIFDPVTGTTHRIEDVVDGRKPIHVVAAAKDGTLLARPVVSWFDQGTRDVIGLRIAGGAIVWATPDHKVLTEYGWRAAGELRKGDRVAGPGGSGNSLALSLTADQMVSALLDAEPPILYSEYDPTSPFSEASMMGLLTNLADRELVHMINWAKRVPGFVDLTLHDQAHLLERAWLEILMIGLVWRSMEHPGKLLFAPNLLLDRNQGKCVEGMVEIFDMLLATSSRFRMMNLQGEEFVCLKSIILLNSGVYTFLSSTLKSLEEKDHIHRALDKITDTLIHLMAKAGLTLQQQHQRLAQLLLILSHIRHMSNKGMEHLYSMKYKNVVPLYDLLLEMLDAHRLHAGGSGASRVQAFADALDDKFLHDMLAEGLRYSVIREVLPTRRARTFDLEVEELHTLVAEGVVVHNCDNKYHDLENGRLIVNIKASKLNFVDIIENKLDKIIEKIDKLDLDKYRFINATNLEHDIKCYYKGFKTKKEVI
**Intein-AcrIIA5(S87)**	MAYGKSRYNSYRKRSFNRSNKQRREYAQEMDRLEKAFENLDGWYLSSMKDSAYKDFGKYEIRLSNHSADNKYHDLENGRLIVNIKACLAEGTRIFDPVTGTTHRIEDVVDGRKPIHVVAAAKDGTLLARPVVSWFDQGTRDVIGLRIAGGAIVWATPDHKVLTEYGWRAAGELRKGDRVAGPGGSGNSLALSLTADQMVSALLDAEPPILYSEYDPTSPFSEASMMGLLTNLADRELVHMINWAKRVPGFVDLTLHDQAHLLERAWLEILMIGLVWRSMEHPGKLLFAPNLLLDRNQGKCVEGMVEIFDMLLATSSRFRMMNLQGEEFVCLKSIILLNSGVYTFLSSTLKSLEEKDHIHRALDKITDTLIHLMAKAGLTLQQQHQRLAQLLLILSHIRHMSNKGMEHLYSMKYKNVVPLYDLLLEMLDAHRLHAGGSGASRVQAFADALDDKFLHDMLAEGLRYSVIREVLPTRRARTFDLEVEELHTLVAEGVVVHNCKLNFVDIIENKLDKIIEKIDKLDLDKYRFINATNLEHDIKCYYKGFKTKKEVI
**Intein-AcrIIA25.1(S30)**	MKNGHMILGQRWTNAIRNETGTSSKMFNLCLAEGTRIFDPVTGTTHRIEDVVDGRKPIHVVAAAKDGTLLARPVVSWFDQGTRDVIGLRIAGGAIVWATPDHKVLTEYGWRAAGELRKGDRVAGPGGSGNSLALSLTADQMVSALLDAEPPILYSEYDPTSPFSEASMMGLLTNLADRELVHMINWAKRVPGFVDLTLHDQAHLLERAWLEILMIGLVWRSMEHPGKLLFAPNLLLDRNQGKCVEGMVEIFDMLLATSSRFRMMNLQGEEFVCLKSIILLNSGVYTFLSSTLKSLEEKDHIHRALDKITDTLIHLMAKAGLTLQQQHQRLAQLLLILSHIRHMSNKGMEHLYSMKYKNVVPLYDLLLEMLDAHRLHAGGSGASRVQAFADALDDKFLHDMLAEGLRYSVIREVLPTRRARTFDLEVEELHTLVAEGVVVHNCKRLYDFKDNNLREIHEALYGLLRAGYDISNMRDVEELAKYVDVKKSHGKLLDVTRDDIELYHRLFVARFGK
**Intein-AcrIIA25.1(S59)**	MKNGHMILGQRWTNAIRNETGTSSKMFNLSKRLYDFKDNNLREIHEALYGLLRAGYDICLAEGTRIFDPVTGTTHRIEDVVDGRKPIHVVAAAKDGTLLARPVVSWFDQGTRDVIGLRIAGGAIVWATPDHKVLTEYGWRAAGELRKGDRVAGPGGSGNSLALSLTADQMVSALLDAEPPILYSEYDPTSPFSEASMMGLLTNLADRELVHMINWAKRVPGFVDLTLHDQAHLLERAWLEILMIGLVWRSMEHPGKLLFAPNLLLDRNQGKCVEGMVEIFDMLLATSSRFRMMNLQGEEFVCLKSIILLNSGVYTFLSSTLKSLEEKDHIHRALDKITDTLIHLMAKAGLTLQQQHQRLAQLLLILSHIRHMSNKGMEHLYSMKYKNVVPLYDLLLEMLDAHRLHAGGSGASRVQAFADALDDKFLHDMLAEGLRYSVIREVLPTRRARTFDLEVEELHTLVAEGVVVHNCNMRDVEELAKYVDVKKSHGKLLDVTRDDIELYHRLFVARFGK

**iA25.1**<br>**(Intein-AcrIIA25.1(S76))**	MKNGHMILGQRWTNAIRNETGTSSKMFNLSKRLYDFKDNNLREIHEALYGLLRAGYDISNMRDVEELAKYVDVKKCLAEGTRIFDPVTGTTHRIEDVVDGRKPIHVVAAAKDGTLLARPVVSWFDQGTRDVIGLRIAGGAIVWATPDHKVLTEYGWRAAGELRKGDRVAGPGGSGNSLALSLTADQMVSALLDAEPPILYSEYDPTSPFSEASMMGLLTNLADRELVHMINWAKRVPGFVDLTLHDQAHLLERAWLEILMIGLVWRSMEHPGKLLFAPNLLLDRNQGKCVEGMVEIFDMLLATSSRFRMMNLQGEEFVCLKSIILLNSGVYTFLSSTLKSLEEKDHIHRALDKITDTLIHLMAKAGLTLQQQHQRLAQLLLILSHIRHMSNKGMEHLYSMKYKNVVPLYDLLLEMLDAHRLHAGGSGASRVQAFADALDDKFLHDMLAEGLRYSVIREVLPTRRARTFDLEVEELHTLVAEGVVVHNCHGKLLDVTRDDIELYHRLFVARFGK
**Intein-AcrIIA32.1(T24)**	MKNEAGKVIVSKSQYANLIRHARCLAEGTRIFDPVTGTTHRIEDVVDGRKPIHVVAAAKDGTLLARPVVSWFDQGTRDVIGLRIAGGAIVWATPDHKVLTEYGWRAAGELRKGDRVAGPGGSGNSLALSLTADQMVSALLDAEPPILYSEYDPTSPFSEASMMGLLTNLADRELVHMINWAKRVPGFVDLTLHDQAHLLERAWLEILMIGLVWRSMEHPGKLLFAPNLLLDRNQGKCVEGMVEIFDMLLATSSRFRMMNLQGEEFVCLKSIILLNSGVYTFLSSTLKSLEEKDHIHRALDKITDTLIHLMAKAGLTLQQQHQRLAQLLLILSHIRHMSNKGMEHLYSMKYKNVVPLYDLLLEMLDAHRLHAGGSGASRVQAFADALDDKFLHDMLAEGLRYSVIREVLPTRRARTFDLEVEELHTLVAEGVVVHNCVEAFKDEFNRITYYDTLTTEASERKRLRIAEHEYKFRVSMQKELHGTEAEITKDFQSVLDYIQEQLKVIVK
**iA32.1**<br>**(Intein-AcrIIA32.1(T40))**	MKNEAGKVIVSKSQYANLIRHARTVEAFKDEFNRITYYDCLAEGTRIFDPVTGTTHRIEDVVDGRKPIHVVAAAKDGTLLARPVVSWFDQGTRDVIGLRIAGGAIVWATPDHKVLTEYGWRAAGELRKGDRVAGPGGSGNSLALSLTADQMVSALLDAEPPILYSEYDPTSPFSEASMMGLLTNLADRELVHMINWAKRVPGFVDLTLHDQAHLLERAWLEILMIGLVWRSMEHPGKLLFAPNLLLDRNQGKCVEGMVEIFDMLLATSSRFRMMNLQGEEFVCLKSIILLNSGVYTFLSSTLKSLEEKDHIHRALDKITDTLIHLMAKAGLTLQQQHQRLAQLLLILSHIRHMSNKGMEHLYSMKYKNVVPLYDLLLEMLDAHRLHAGGSGASRVQAFADALDDKFLHDMLAEGLRYSVIREVLPTRRARTFDLEVEELHTLVAEGVVVHNCLTTEASERKRLRIAEHEYKFRVSMQKELHGTEAEITKDFQSVLDYIQEQLKVIVK
**Intein-AcrIIA32.1(A73)**	MKNEAGKVIVSKSQYANLIRHARTVEAFKDEFNRITYYDTLTTEASERKRLRIAEHEYKFRVSMQKELHGTECLAEGTRIFDPVTGTTHRIEDVVDGRKPIHVVAAAKDGTLLARPVVSWFDQGTRDVIGLRIAGGAIVWATPDHKVLTEYGWRAAGELRKGDRVAGPGGSGNSLALSLTADQMVSALLDAEPPILYSEYDPTSPFSEASMMGLLTNLADRELVHMINWAKRVPGFVDLTLHDQAHLLERAWLEILMIGLVWRSMEHPGKLLFAPNLLLDRNQGKCVEGMVEIFDMLLATSSRFRMMNLQGEEFVCLKSIILLNSGVYTFLSSTLKSLEEKDHIHRALDKITDTLIHLMAKAGLTLQQQHQRLAQLLLILSHIRHMSNKGMEHLYSMKYKNVVPLYDLLLEMLDAHRLHAGGSGASRVQAFADALDDKFLHDMLAEGLRYSVIREVLPTRRARTFDLEVEELHTLVAEGVVVHNCEITKDFQSVLDYIQEQLKVIVK
